# Investigating the Theranostic Potential of Graphene Quantum Dots in Alzheimer’s Disease

**DOI:** 10.3390/ijms24119476

**Published:** 2023-05-30

**Authors:** Max Walton-Raaby, Riley Woods, Subha Kalyaanamoorthy

**Affiliations:** Department of Chemistry, University of Waterloo, Waterloo, ON N2L 3G1, Canada; max.walton-raaby@uwaterloo.ca (M.W.-R.); rt3woods@uwaterloo.ca (R.W.)

**Keywords:** Alzheimer’s disease, Tau protein, nanomedicine, theranostic, graphene quantum dots, neurofibrillary tangles

## Abstract

Alzheimer’s disease (AD) is one of the leading causes of death worldwide, with no definitive diagnosis or known cure. The aggregation of Tau protein into neurofibrillary tangles (NFTs), which contain straight filaments (SFs) and paired helical filaments (PHFs), is a major hallmark of AD. Graphene quantum dots (GQDs) are a type of nanomaterial that combat many of the small-molecule therapeutic challenges in AD and have shown promise in similar pathologies. In this study, two sizes of GQDs, GQD7 and GQD28, were docked to various forms of Tau monomers, SFs, and PHFs. From the favorable docked poses, we simulated each system for at least 300 ns and calculated the free energies of binding. We observed a clear preference for GQD28 in the PHF6 (^306^VQIVYK^311^) pathological hexapeptide region of monomeric Tau, while GQD7 targeted both the PHF6 and PHF6* (^275^VQIINK^280^) pathological hexapeptide regions. In SFs, GQD28 had a high affinity for a binding site that is available in AD but not in other common tauopathies, while GQD7 behaved promiscuously. In PHFs, GQD28 interacted strongly near the protofibril interface at the putative disaggregation site for epigallocatechin-3-gallate, and GQD7 largely interacted with PHF6. Our analyses revealed several key GQD binding sites that may be used for detecting, preventing, and disassembling the Tau aggregates in AD.

## 1. Introduction

Tauopathies are neurological disorders characterized by the misfolding and aberrant aggregation of the microtubule-associated protein Tau [1,2,3]. They encompass a broad range of neurodegenerative diseases, including but not limited to frontotemporal dementia, characterized by frontotemporal lobar degeneration; Pick’s disease, characterized by Pick cells and bodies; progressive supranuclear palsy 1, characterized by neurofibrillary tangles (NFTs) and gliosis; and Alzheimer’s disease (AD), a hallmark neurodegenerative disease characterized by the misfolding and aggregation of a wide range of intrinsically disordered proteins (IDPs), including Tau [4,5,6,7]. The burden of such neurodegenerative diseases on our healthcare systems and society is only increasing and is predicted to reach a staggering 152.8 million cases by 2050 [8], a fourfold increase from 2015 [7]. Furthermore, AD is the most common neurodegenerative disease, being one of the leading causes of death worldwide. The design and development of new diagnostic and therapeutic tools targeting Tau is a promising strategy to treat AD.

**Figure 1:**
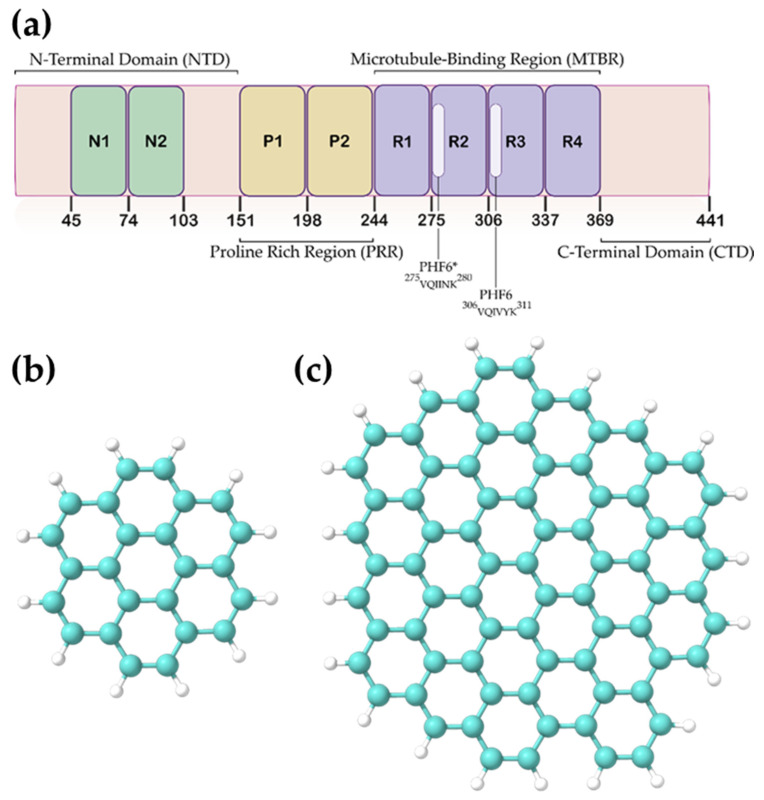
(**a**) Sequence and domain organization of 2N4R Tau protein and the graphene quantum dots (GQDs) used in this study: (**b**) GQD7, (**c**) GQD28.

To rationally design AD therapeutics, an intimate understanding of the physiological and pathological functions of Tau is required. Tau was first discovered in 1975 when Weingarten et al. identified a protein contaminant that co-purified with microtubules. Upon further investigation, this contaminant, now known as the microtubule-associated protein Tau, was found to be critical for microtubule stability [9]. Tau is encoded by the microtubule-associated protein Tau (MAPT) gene located on chromosome 17. Its sequence can be divided into four distinct regions (Figure 1): the N-terminal domain (NTD), the proline-rich region (PRR), the microtubule-binding region (MTBR), and the C-terminal domain (CTD). From the alternative splicing of the MAPT gene, six isoforms of Tau are produced ranging from 352–441 amino acids in length. These isoforms are distinguished by containing either zero, one, or two N-terminal inserts (0N, 1N, 2N) and three or four microtubule-binding repeats (R1, R3, R4 or R1, R2, R3, R4) in the MTBR. The isoforms are referred to as 0N3R, 0N4R, 1N3R, 1N4R, 2N3R, and 2N4R. The inclusion of both the R2 and R3 regions is typically considered essential for aggregation kinetics [10,11]. On the other hand, a recent article demonstrated that the exclusion of the R2 region may increase the rate of aggregation [12].

The onset and propagation of AD involves the formation of misfolded and oligomerized Tau and the appearance of NFTs [13]. The major components of tangles are paired helical filaments (PHFs, Figure S1) and straight filaments (SFs, Figure S1), composed primarily of 3R and 4R isoforms. In the microtubule-binding region, at least two hexapeptide segments are known to be involved in Tau aggregation [14]. These hexapeptides are PHF6* (^275^VQIINK^280^) and PHF6 (^306^VQIVYK^311^), present in R2 and R3 regions, respectively. Enriched with hydrophobic residues, these hexapeptides facilitate intermolecular interactions essential for the formation of the characteristic β-sheet structures in NFTs [13]. Although mechanistic insights are crude and lacking in experimental studies, previous in silico work, including molecular dynamics (MD) simulations, has proven to be a powerful tool in facilitating such discoveries. In particular, the development of enhanced sampling and analysis techniques enables the study of these systems, uncovering the mechanistic and kinetic importance of certain regions on Tau in the aggregation process, and guiding future therapeutic targets [15,16,17].

Currently, most of the available therapies for tauopathies such as AD approved by regulatory agencies like the United States Food and Drugs Administration (FDA), European Medicines Agency, and Pharmaceuticals and Medical Devices Agency employ the use of acetylcholine esterase inhibitors and *N*-methyl-D-aspartate receptor antagonists to treat mild to severe AD [13]. While these treatments struggle to block the progression of the disease, they can temporarily increase cognitive functions through partial amelioration of cholinergic and glutamatergic neurotransmission [18]. Even though these classes of drugs can slow the onset and even reduce dementia-related symptoms, they are by no means standalone cures. Since Tau is an essential protein for neuronal survival, any new therapy must not treat the conditions by blocking or eliminating Tau, as this could be more detrimental to the organism than the tauopathy itself. Hence, there is a need for an intimate understanding of the structural and mechanistic features that lead to the formation of NFTs. The application of AD therapeutics is typically limited due to their instability in circulation, inconsistent uptake, and low bioavailability, caused by the limited permeability of the blood–brain barrier (BBB). In fact, over 100 drugs that had billions of dollars invested in their development between 2010 and 2015 have either been abandoned or have failed clinical trials, mainly failing to reach efficacy endpoints [19]. These drugs include monoclonal antibodies, gamma-secretase inhibitors, dimebon, neurochemical enhancers, and a few Tau drugs aimed at regulating its hyper-phosphorylation and subsequent aggregation [19,20]. However, it should be mentioned that there have been recent developments that have allowed antibodies to cross the BBB, potentially enabling highly specific therapeutics [21]. Nevertheless, future AD therapeutics must focus on new methods and materials to pass drugs through the BBB. These methods will also allow AD diagnostics to reach their targets.

Nanoparticles (NPs) are a vast class of molecules that show great promise in crossing the BBB as well as in delivering drugs into the brain [22]. NPs have high biocompatibility due to their highly ordered, stable, polymeric structures. One class of NPs that has generated much interest across several fields of medicine is graphene quantum dots (GQDs). More specifically, they have garnered interest due to their unique electrical and photochemical properties; being a zero-dimensional nanomaterial with a lateral size of less than 100 nm, they possess low toxicity and high fluorescent activity, properties which can be fine-tuned by simply changing particle size; as well as high chemical inertness, and excellent photostability, granting them superior storage capabilities for longer-term use compared to other quantum dots and carbon-based nanomaterials [23,24]. Furthermore, GQDs are highly versatile in terms of functionalization. For example, GQDs can be “doped” within their graphene scaffold with heteroatoms such as N, O, and F, and their edges can be functionalized with several groups as well. Additionally, their surfaces can be functionalized with small peptides, nucleic acids, and various polymers [25,26,27,28,29,30]. Currently, it is highly challenging to attain site-specific GQD functionalization experimentally, limiting the systematic progression of GQD design and hindering direct comparisons to theoretical work. Nevertheless, these modifications may be tuned to increase the binding affinity and specificity, as well as to alter GQD absorption spectra, guiding the rational design of GQDs for the detection and prevention of AD [31].

Over the last decade, numerous studies have evaluated the potential of GQDs and some of their functionalized forms in the modulation of amyloidogenic aggregation, another key target of AD pathology [13,24,26,32,33]. For example, Yousaf et al. reported that F-doped GQDs inhibited human islet amyloid polypeptide (HIAPP), a protein used to model the aggregation behavior of amyloid at a rate of inhibition of 95% [26]. Kim et al. demonstrated GQD passage through the BBB and observed the aggregation prevention and defibrillation of α-synuclein [34]. Lui et al. demonstrated the positive regulation of amyloid-β (Aβ_1-42_) in vitro and made the case that GQDs have great potential in maintaining amyloid aggregates in their non-toxic state and could eventually be widely used as a functional nanomaterial in protein amyloidogenesis [24]. Given that these previously reported uses of GQDs inhibit a similar pathological mechanism of aggregation as Tau undergoes, we hypothesize that GQDs may also show promise in preventing Tau aggregation.

The goal of this work was to determine how GQDs and various AD-related forms of Tau interact. First, we identified various GQD binding sites of AD Tau and its aggregates through exhaustive docking. Second, we evaluated the stabilities, binding affinities, and conformational dynamics of these docked poses through MD simulations. This process determined whether GQDs are selective to any of the tested Tau structures (monomeric, PHF, and SF) or binding sites, identified key interacting residues, and provided insights into the structural impacts of GQD–Tau interactions. By comparing two varied sizes of GQDs, we also observed how GQD size affects the strength and location of interactions. The findings of this work enhance our current understanding of how Tau protein and GQDs could interact and may help to establish a novel theranostic for AD.

## 2. Results and Discussion

### 2.1. Survey of Available Structures of Tau Protein

The availability of Tau structures in the context of Alzheimer’s disease is still quite limited, despite recent monumental contributions [35]. We collected the structures of many regions of Tau, with a particular focus on the MTBR (see Table S1 for a complete list of structures used). R2 and R3 were the focus of this study given their suspected roles in AD pathology, comprising the PHF6* and PHF6 hexapeptide regions, and have many available structures. On the other hand, the NTD, PRR, and CTD have fewer available structures; hence, conclusive results would be hard to obtain for these regions.

Many monomeric structures of Tau were chosen from various sources. Although the chosen structures could not cover all conformations of monomeric Tau, they were deemed representative structures for the docking analysis and subsequent MD analysis. Since most structures were resolved in unnatural conditions (e.g., complexed with aggregation-inducing agents, bound to antibodies), it can be expected that the initial conformations of these structures will affect the results of the docking study. Therefore, we used molecular dynamics simulations to account for at least some, if not all, of the dynamics and conformational changes of these systems.

### 2.2. Docking of GQD7 and GQD28 to Tau Monomers and Aggregates

**Figure 2:**
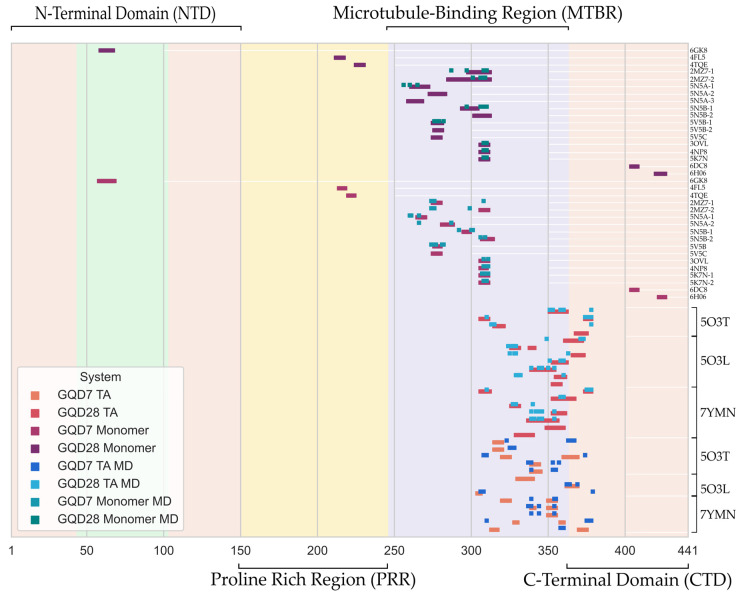
Docked regions of 2N4R Tau protein’s sequence relative to the strongest interacting residues following molecular dynamics (MD) simulations. Residue numbers are given in the *x*-axis and the PDBIDs of the Tau protein used for docking (monomeric and Tau aggregate (TA)) and MD simulations are given in the *y*-axis.

All of the docked poses tested in this study are displayed in Figure 2, including poses that had positive docking scores. From this figure, it can be seen that the GQDs had a clear preference for the MTBR and especially showed favorable binding to R2 and R3 regions in the monomeric Tau and the R3 and R4 regions in the Tau aggregates. Since the aggregated structures of Tau lacked the R2 region, it was not possible to characterize the binding affinity of GQDs in the aggregated R2 conformations. Further, it is well known that most docking scoring functions are biased towards providing lower energy (better docking) scores for larger ligands. To address this inherent bias, we focused on the relative ranking of the poses rather than the magnitudes of the docking scores of the GQDs.

Docking GQD28 to the selected monomeric structures of Tau produced many negative docking scores in the MTBR (Table 1), indicating potential binding to this region. Specifically, GQD28 favored monomeric structures that included the PHF6 hexapeptide region and displayed unfavorable interactions when docked to the PHF6* hexapeptide region. In the case of 2MZ7 (residues 267–312), none of the docking poses centered on PHF6* produced negative docking scores. Instead, two poses were produced: the first pose displayed interactions with residues, K298, Q307, I308, V309, and Y310 and had a docking score of −0.60 kcal/mol, while the second pose showed interactions with I297, H299, P301, G303, and K311, with a similar score of −0.63 kcal/mol. In 5N5B, the other long monomer covering the PHF6 hexapeptide region (residues 292–312), GQD28 docked to a similar region as the second pose in 2MZ7. In this structure, the interactions were with nearly every residue between 293 and 301. When docking to only the PHF6 hexapeptide (PDBIDs 3OVL, 4NP8, and 5K7N), a large variation in the docking scores was observed (−0.53, −1.38, and −1.07 kcal/mol, respectively) due to the varied interactions formed by different conformations. Interestingly, GQD28 was near to residues I308, V309, and Y310 in these favorable binding poses. Aside from PHF6 and its surrounding residues, one negative docking score was produced preceding the PHF6* hexapeptide region (PDBID 5N5A). In this structure, GQD28 interacted most with E264, K267, H268, and Q269.

Similar to GQD28, GQD7 produced nearly exclusively negative docking scores in monomeric Tau structures containing the MTBR region of the Tau sequence. All but one pose outside the MTBR, PDBID: 6DC8 (Table 2) produced positive docking scores. On the other hand, GQD7 obtained favorable docking scores to both PHF6* and PHF6. For example, GQD7 docking to 2MZ7 produced two negative scores resulting in interactions with I277, I278, N279 and I308, V309, Y310, with affinities of −1.40 kcal/mol and −1.53 kcal/mol, respectively. Further docking to the PHF6*-containing monomeric structure 5N5A produced two poses which interacted with non-PHF6* hydrophobic residues (I260, G261, L266, and V287). Docking to the PHF6-containing monomeric structure 5N5B obtained two negative scores with one interacting specifically with PHF6 residues V306 and V309. When docked to PHF6, GQD7 obtained more negative docking scores than when docked to PHF6*. Indeed, docking GQD7 solely to PHF6* hexapeptide (5V5B, 5V5C) produced only positive docking scores, whereas docking to solely PHF6 hexapeptide (3OVL, 4NP8, 5K7N) produced the most negative docking scores (−1.51, −1.22, −1.71, and −1.51 kcal/mol, respectively) and caused it to interact most frequently with I308, Y310 and K311. These results suggest that PHF6* interaction depends on the presence of both hexapeptides, and the non-PHF6* residues are likely to play a key role in binding GQDs, as seen with 5N5A.

GQD28 was docked along the entire sequence of each Tau aggregate. The docked poses with negative docking scores are shown in Figures S2–S4. In the SFs, GQD28 identified five poses with negative docking scores in PDBID 6HRF, while only three poses with negative docking scores were identified in PDBID 5O3T (Table 3). Pose 1 in 5O3T and Pose 2 in 6HRF were nearly identical in their interactions and produced docking scores of −4.46 and −3.21 kcal/mol, respectively (Table S2). The difference in docking scores is due to the increased number of interacting chains in 5O3T (five chains), compared to 6HRF (three chains), which is seen throughout the study for both SF and PHF systems. In both poses, S352, K353, I360, and H362 were found to be the strongly interacting residues. Another similar pair of docking poses was achieved in 5O3T Pose 2 and 6HRF Pose 3, with docking scores of −3.13 and −3.21 kcal/mol, respectively. In this binding site in 5O3T, GQD28 interacted most with residues 374 to 378 and 306 to 310, while in 6HRF, GQD28 interacted most with residues 374 to 380 and 308 to 311 (Figure S4). This binding site is particularly interesting since GQD28 forms similar interactions with the PHF6 hexapeptide region in monomeric Tau. GQD28 also identified many other single-chain sites in 6HRF, producing docking scores between −1.52 and −0.87 kcal/mol.

Exploration of the same structures with GQD7 produced more poses compared to GQD28, with five and eight poses obtained for 5O3T and 6HRF, respectively. The smaller size of GQD7 allowed it to adopt multiple poses in different regions when docked to the SF structures and resulted in the two best scoring poses (Poses 2 and 3) presenting in 5O3T, with −2.38 and −2.41 kcal/mol, respectively. The 6HRF structure resulted in a pose with −2.25 kcal/mol (Table S3, Pose 3). In the similar poses (Poses 1 and 2 in 5O3T and Pose 3 in 6HRF), GQD7 formed interactions with all residues in the 315–320 region of both structures and in the 320–325 and 360–365 regions of 5O3T. Similar interactions can be seen in 6HRF with less affinity (Poses 4, 5, 6, and 7), further supporting the favorable binding of GQD7 to this region in SFs. The remaining poses obtained mediocre scores along the rest of the sequence of 6HRF, except for Pose 1, which had a score of −2.23 kcal/mol and interacted with residues 304–308 and 380, a similar region to GQD28.

In the case of the PHFs, PDBIDs 5O3L and 7YMN, GQD28 was identified to have multiple favorable binding modes with six and five negative docking scores, respectively. In 5O3L, GQD28 had the most negative docking score in this study (–4.74 kcal/mol) in Pose 4 (Table 3). In this pose, GQD28 exhibited a slightly different binding mode from the SFs (GQD28 Pose 1 in 5O3T) by interacting with residues Q351, S352, K353, H362, and K369. GQD28 also identified the same binding site in 7YMN (Pose 2) with nearly identical interactions, though its docking score was much weaker (–1.28 kcal/mol). A similar single-chain interaction was observed in Poses 6 and 5 in 5O3L and 7YMN, producing docking scores of −1.83 and −1.85 kcal/mol, respectively. GQD28 interacted most with residues 330–334 and 357–361 in 5O3L, and with residues 337–346 and 350–355 in 7YMN. The last similar binding sites worth mentioning are Pose 2 in 5O3L and Pose 3 in 7YMN, which produced docking scores of −1.84 and −1.42 kcal/mol, respectively. In both cases, GQD28 primarily interacted with N327, H329, K331, E338, and K340 through Pi–Pi stacking and cation–Pi interactions.

The docking of GQD7 along the sequence of the PHF structures produced six poses in 7YMN but only two poses in 5O3L. The two 5O3L poses obtained similar scores of −1.78 and −1.70 kcal/mol (Table 4), forming interactions with residues 330–340 and 362–369, respectively. Given the fact that a large difference in the number of poses was observed between the two PHF structures, it is still promising that 7YMN produced its best pose in a region similar to that of the 5O3L poses. Specifically, Pose 5 obtained a score of −2.49 kcal/mol, interacting with residues 328–330 and 358–360. Like GQD28, GQD7 also obtained negative docking scores in the 350–355 region of 7YMN.

Through exhaustive docking, several putative binding sites were identified in both monomeric and aggregated Tau structures. For monomeric Tau, GQD28 formed most of its interactions with residues I308, V309, and Y310 from the PHF6 hexapeptide region and some interactions with residues before and after the PHF6* hexapeptide region. GQD7 interacted similarly with PHF6; however, it also demonstrated binding sites with residues 260–266, as well as 277–279. In SFs, GQD28 bound most strongly to the C-shaped curve of the SF (residues 351–360) and the termini of the fibrils. In PHFs, GQD28 also identified the C-shaped binding site in the PHFs and a binding site between the protofibrils (residues 326–331 and 338–341). In SFs, GQD7 identified many binding sites; however, the lowest docking scores were mainly at the binding site between the protofibrils, supporting the GQDs binding to this region in PHFs.

### 2.3. Structural Stability of the Tau Monomers and Aggregates

In the following sections, we investigate the dynamics of the protein and protein–GQD systems. Compared to static simulations like docking, MD allows for Tau to explore various energetically accessible conformations. Consequently, the impact of GQD binding on the dynamics, secondary structure, and stability of Tau can be determined. For this, we utilize root-mean-square deviation (RMSD) and the dictionary of secondary structures (DSSP) analyses available in the AMBER package. For all systems, simulations were run for 300 to 340 ns.

The monomeric structures were relatively unstable during the MD simulations when unbound to the GQDs. For instance, the unbound simulation of monomeric hexapeptide (PDBID: 5K7N) and a 46-residue long peptide (PDBID: 2MZ7) exhibited increased fluctuations, with an increase in the peptide length. This is unsurprising since Tau is intrinsically disordered, with many metastable conformational states. The 5K7N system explored many different conformations and produced an average RMSD value of 2.82 ± 0.81 Å during the last 100 ns of the simulation. On the other hand, the longer peptide, the 2MZ7 monomer structure of Tau, displayed higher fluctuations with an average RMSD of 11.02 ± 1.10 Å. While the increased length of the monomers enabled additional interactions between different residues on the peptide, thereby allowing the formation of some defined secondary structures (e.g., alpha helices, bends, and turns), the overall flexibility of the system was high, re-emphasizing the intrinsically disordered nature of the Tau.

**Figure 3:**
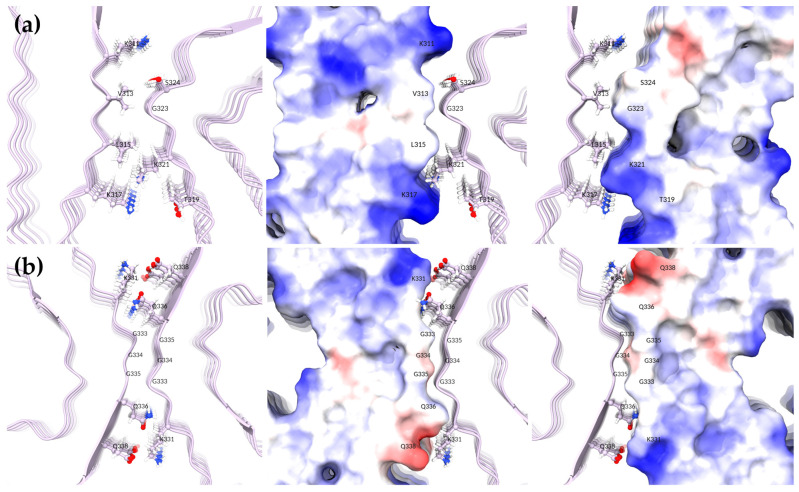
Comparison of protofibril interfaces between (**a**) a straight filament (SF) (5O3T) and (**b**) a paired helical filament (PHF) (5O3L). The middle and rightmost figures are displayed as electrostatic surfaces.

In addition to the monomeric forms, for each Tau aggregate, a protein-only system was simulated to determine the stability of the protein in the absence of the GQDs. Unsurprisingly, the structures with ten chains (PDBIDs 5O3T and 5O3L) were much more stable throughout the MD simulations than the six-chain systems (PDBIDs 6HRF and 7YMN), which twisted and displayed higher chain mobility. For 6HRF, a SF, both trimeric protofilaments became dissociated in all systems studied, including the protein-only system; hence, the MD results of 6HRF were excluded. Interestingly, this was not the case for 7YMN, a PHF structure. When comparing the protofibril interfaces between SFs and PHFs, it can be noted that PHFs form many more compatible electrostatic and hydrophobic interactions between protofibrils (Figure 3). The protofibrils in SFs are held together mainly by the hydrophobic interactions of V313 and L315 with neighboring glycine residues. Interestingly, K317 and K321 also form electrostatically repulsive interactions in SFs. On the other hand, PHF protofibrils are held together by much more compatible electrostatic interactions between K331 and E338 on both sides of the interface, as well as several hydrophobic interactions between glycine residues. We suspect that the decreased number of chains in 6HRF, relative to the larger SF (5O3T), along with the twisting of the protofibrils, further destabilized the already weak protofibril interface.

Comparing the stabilities of 5O3T (SF) and 5O3L (PHF) in the RMSDs, 5O3T exhibits higher stability, with an average RMSD of 1.80 ± 0.09 Å, when compared to 5O3L (RMSD 4.05 ± 0.98 Å) (Figures S5–S8). This is surprising given the weaker interfacial interactions observed in the SF. Nevertheless, 5O3T remained rigid during the simulation, while 5O3L adopted a conformation where the protofibrils were slightly twisted out of plane. From the secondary structure plots (Figures S9 and S10), it can be noted that both 5O3T and 5O3L maintain nearly all of their characteristic beta sheets throughout the length of the simulation, indicating that this simulation timescale was sufficient for proper system stabilization. In general, the only consistent structural change was a slight loss of beta sheet stacking at the termini. A similar trend was observed in the 7YMN PHF structure, which exhibited an average RMSD of 6.35 ± 0.54 Å.

### 2.4. Structural Stability of the Tau Monomers and Aggregates in the Presence of GQDs

The binding of monomeric Tau to either GQD induces more rigidity in the tau peptides. When bound to GQD28, 5K7N formed bends and turns which were not present in the protein-only system. In the case of 2MZ7, GQD28 binding (Pose 1) resulted in a local conformation that included more turns and bends which the alpha helix present in the protein-only simulation was not capable of forming (Figure S11). In addition, the residues that were strongly interacting with GQD28 lost nearly all of their helical character, as seen by plotting the occurrence of secondary structures throughout the trajectory (Figure S12). Monomeric Tau bound to GQD28 tends to lose helicity and favor more bent and turn secondary structures. A similar trend was observed with GQD7. While the secondary structural changes in the Tau monomer upon binding GQD can be seen from the classical MD simulations, its implication on the aggregation mechanisms remains inconclusive. Nevertheless, the RMSD and secondary structure analyses together (see Figures S13 and S14) illustrate that GQD binding can alter the conformational landscape and dynamics of the monomer Tau even within the short simulation timescale.

GQDs have a variable effect on the stability of the Tau aggregates; however, they typically retain the binding site predicted by the docking study (Figure 2). In the SF 5O3T, both GQDs had a significant destabilizing effect, resulting in large RMSD oscillations (e.g., 3.41 ± 0.24 and 4.94 ± 0.87 Å, for GQD28 Pose 1 and GQD7 Pose 1, respectively) when compared to the protein-only system (1.80 ± 0.09 Å) (Figures S5 and S6). Moreover, the total beta sheet content in the protein-only system was decreased, with the binding site residues in particular converting to bends and turns (Figures S9 and S15). It is unclear why the GQDs destabilized 5O3T in all the studied systems. It is possible that the protofibril interface in 5O3T is weak and the presence of GQDs causes further destabilization. In the PHF structures, the GQDs had both stabilizing and destabilizing effects. In 5O3L, the GQD-bound systems contained significantly smaller fluctuations, while in 7YMN, the GQD28(7)-bound systems contained equivalent or larger fluctuations than the protein-only systems. In 5O3L, the protein-only RMSD was 4.05 ± 0.98 Å, while the GQD-bound RMSDs were 2.53 ± 0.27 Å and 3.61 ± 0.12 Å for GQD28 (Pose 2) and GQD7 (Pose 2) (Figures S7 and S8), respectively. In contrast, the protein-only RMSD of 7YMN was 6.35 ± 0.54 Å, while the GQD-bound RMSDs were 6.70 ± 1.05 Å and 7.76 ± 0.72 Å for GQD28 (Pose 3) and GQD7 (Pose 1) (Figures S16 and S17), respectively. Given the larger size of GQD28, it is unsurprising to note the significant destabilization of the smaller, more flexible PHF (7YMN). However, the lower standard deviations observed in GQD7 systems suggest the existence of local interactions, which likely confer a small degree of stability while still destabilizing the fibrils. Further, the opposite trends observed between the two PHF structures (5O3L and 7YMN) are likely due to the difference in the size of the aggregates (oligomeric states). Therefore, testing multiple aggregate structures and possibly different GQD functionalization might be needed to accurately predict the impact of GQD binding to PHFs. Although the stability analyses of the two PHFs were different, it is worth noting that both the structures exhibited a decrease in beta sheet content when GQDs were present (Figure S18).

It is hard to predict the long-term behavior of these systems over such short timescales. The stability trends between RMSD and secondary structure analyses were slightly different. For the SF (5O3T), an increase in RMSD fluctuations was associated with decreased beta sheet content and increased occurrence of bends and turns. Conversely, in the PHFs (5O3L and 7YMN), the RMSD trends indicate generally increased stability despite the conversion of some secondary structure from beta sheets to bends and turns. Nevertheless, in both TAs, a decrease in beta sheet content near the binding site was observed. If longer timescales were computationally accessible, it is possible that this loss of beta sheet character would propagate through the fibril, leading to disaggregation, though this is speculative at this point. To assess the stability of Tau–GQD systems, we will be performing subsequent experimental analyses for validation in future work.

### 2.5. Investigating the Binding of GQDs to Monomeric Tau’s Microtubule Binding Region

It is important to understand how GQDs may interact with monomeric Tau to predict their effects in vivo. Due to the limited knowledge of monomeric Tau binding, it is not clear whether binding to monomeric Tau would produce a positive effect in AD. However, due to the prion-like mechanism of Tau aggregation, we argue that binding to monomeric Tau could prevent its aggregation. The wrapping of monomeric Tau around GQDs may prevent the necessary level of contact for aggregation with the oligomers and aggregates. Furthermore, given that most Tau is bound to the microtubule rather than dispersed in the axonal cytoplasm, capturing monomeric Tau is not expected to alter its physiological functions.

The interactions between GQD28 and monomeric Tau were dominated by Van der Waals (VdW) forces. Using the longest monomeric Tau sequence, PDBID 2MZ7, two unique poses were pursued for MD analyses. The first pose achieved a (MM/PBSA) BFE (binding free energy) of −26.37 ± 4.83 kcal/mol (Table 1), which indicates that this pose has a notable affinity to GQD28 (Figure S19). The first pose was centered on the PHF6 region, interacting most strongly with Y310 through Pi stacking interactions, followed by I308 and V309 through hydrophobic interactions (Figure S20). In addition, V287 and I297 contributed less through hydrophobic interactions. In this pose, GQD28 interacted with Tau in similar regions as epigallocatechin-3-gallate (EGCG), a molecule that prevents Tau aggregate maturation (potentially by capturing monomeric Tau); the interactions in an elongated model of 2MZ7 were with H299, Y310, P312, among others [36]. The second docked pose had a smaller BFE of −17.54 ± 3.10 kcal/mol. GQD28 had weaker interactions in this pose, interacting with P301, V306, I308, and V309. It is unclear why GQD28 was incapable of interacting with both hexapeptide regions. Given that monomeric Tau wraps around GQDs, the size of GQD28 may prevent contact with both hexapeptide regions simultaneously. Nevertheless, GQD28 binds strongly to the PHF6 region of Tau, which is therapeutically promising.

To further explore GQD28′s interactions with the PHF6 region, we used a monomeric structure of Tau covering residues 292–319 (PDBID 5N5B). This structure had a BFE of −16.62 ± 2.84 kcal/mol and had similar interactions to the second docked pose of 2MZ7. GQD28 formed strong interactions with I297, V306, I308, and Y310 (Figure S20). Equivalent results were obtained with three smaller structures (PDBIDs 3OVL, 4NP8, and 5K7N), with GQD28 interacting most strongly with I308, V309, and Y310 in each case (Figure S20). GQD28′s consistent binding to this region could prevent Tau monomers from undergoing template-induced aggregation in the presence of Tau protofilaments.

To check whether GQD28 could bind to the PHF6* region, a monomeric structure of Tau, covering residues 254–290 (PDBID 5N5A), was simulated. An MM/PBSA BFE of −13.16 ± 4.18 kcal/mol was obtained, with GQD28′s interactions preceding the PHF6* region: V256, I260, and N265 (Figure S20). As a control for our docking screening method, we ran an MD simulation for 5V5B (Pose 1), which had a docking score of 1.10 kcal/mol. GQD28 did not interact with any of the residues strongly; hence, the protein explored many different GQD binding poses. Surprisingly, 5V5B still produced a BFE of −9.91 ± 3.18 kcal/mol, demonstrating polar interactions with Q276 and N279 and hydrophobic interactions with I278 and L282 (Figure S20). GQD28 may interact with the PHF6* region; however, GQD28 is expected to preferentially bind to the PHF6 region.

Simulating the 2MZ7 structure allowed the exploration of GQD7 binding to both PHF6 and PHF6*. Two docking poses were pursued with MD that showed stable GQD binding and allowed for appropriate MM/PBSA analysis. The first pose obtained a BFE of −12.76 ± 3.75 kcal/mol, centered on residues K274, Q276 and I308 (Table 2). Throughout the trajectory, GQD7 formed the strongest interaction with I308, supported by the high VdW contribution to the total BFE (Figure S21). Over this, GQD7 interacted simultaneously with the PHF6* residues K274 and Q276 through cation–Pi interactions. The second pose showed similar binding residues and obtained a BFE of −11.13 ± 3.18 kcal/mol. In this case, GQD7 interacted strongly with residues K274, Q276, and H299 (Figure S21). The aggregation of PHF6 and PHF6* are known to be important in tauopathies and form parallel beta sheets with one another. These analyses show that GQD7 binds stably to both PHF6 (I308) and PHF6* (K274 and Q276) residues, suggesting that GQD7 may have the potential to prevent the pathological aggregation of these two hexapeptides.

In analyzing PDBID 5N5A, a Tau monomer that only contains the PHF6* hexapeptide region, we pursued two GQD7 poses with negative docking scores. The first was centered around residues preceding PHF6* (residues 265–270) and the second proceeding the PHF6* region (residues 284–288). Both trajectories, however, captured GQD7 translating and interacting strongly with hydrophobic residues I260, G261, L267, and V287 producing BFEs of −15.38 ± 3.70 and −9.40 ± 3.91 kcal/mol, respectively (Figure S21). Additionally, when testing a system that only contained the PHF6 hexapeptide region (PDBID 5N5B), we pursued two poses with negative docking scores. Both displayed similar stable behavior throughout the MD trajectory and produced BFEs of −9.32 ± 4.01 and −12.10 ± 3.53 kcal/mol, respectively. GQD7 interacts most strongly with PHF6 residues V306 and V309, and the hydrophobic valine residues preceding the PHF6 (G292, V300, P301) (Figure S21). Being near I308, one of the key binding residues identified in 2MZ7, it is clear that GQD7 possesses binding affinity for the aggregation-prone PHF6 region of monomeric Tau. Further, our simulation results show that, unlike GQD28, GQD7 can interact with both hexapeptide regions; however, it forms stronger interactions with PHF6.

From observing the various monomeric Tau structures, it is evident that both GQDs interact strongly with monomeric Tau, mainly through VdW interactions. The dominant interactions were observed with residues in and around the PHF6 region, with I308, V309, and Y310 forming consistently strong interactions with both GQDs. GQD28 seems to be much more selective to the PHF6 region, which is considered more pathogenic. On the other hand, GQD7 formed strong interactions with the PHF6 region, which lowered the BFE by nearly 10 kcal/mol when GQD7 formed synergistic interactions with the PHF6* region. From these results, it is suspected that GQDs can bind to monomeric Tau in various conformations. This binding may lock Tau monomers into conformations, preventing their association with Tau oligomers and aggregates, assuming the affinity to the GQD is sufficient.

### 2.6. Investigating GQDs Binding to Tau Aggregates

It is highly desirable for GQD7 and GQD28 to bind to AD Tau aggregate structures since this could prevent the process of aggregation and even disaggregate pre-existing Tau oligomers and aggregates. Additionally, if GQDs bind selectively to either Tau aggregate, they may function as fluorescent biomarkers. Therefore, we studied the unique and favorable poses of GQDs to different aggregated structures of Tau using at least 300 ns molecular dynamics simulation to assess their binding affinities and stabilities. While 300 ns is not sufficient time to observe what large-scale conformational changes occur upon binding such as disaggregation, it is useful to determine binding affinities. The calculated BFEs are listed in Table 3 and Table 4, and their affinities are discussed based on the filament architecture.

#### 2.6.1. Investigating GQDs Binding to Straight Filaments

GQD28 produced approximately the same magnitude as BFEs in SF and PHF structures. For the SF (PDBID 5O3T), the three poses with negative docking scores were pursued in MD simulations. The first pose produced the most negative MM/PBSA BFE in the SF systems of −10.76 ± 4.94 kcal/mol (Table 3), interacting most with I360, as well as with Q351, L353, D358, and F378 (Figure S22). Furthermore, this region showed the highest stability throughout the simulation (Figure S5 and S9). Interestingly, the C-terminal of the first chain reoriented itself entirely to interact with GQD28. This binding site was seen in every Tau aggregate and seemed to be favored by the docking software, which always predicted it to be the most negative docking score. Curiously, this region only produced promising MM/PBSA BFEs in the SF. Nevertheless, this binding site is of particular interest in Tau aggregates since it is unavailable in other common tauopathies such as Pick’s, corticobasal degeneration, and progressive supranuclear palsy [37]. Moreover, this binding site has been concluded via theoretical methods to be the binding site for bTVBT4, a luminescent ligand that can selectively detect AD Tau aggregates over Pick’s fibrils [38]. In the case of bTVBT4, the strongest interactions in the binding site were with I360, T361, and H362, which is quite similar to GQD28. Given GQD28’s selectivity for this site in SFs, it is possible that GQD28 could be used for fluorescent SF detection.

GQD28′s second pose with PDBID 5O3T had a BFE of −4.70 ± 4.43 kcal/mol. GQD28′s main interactions were with Y310, H374, L376, T377, and F378, with the largest contribution from hydrophobic interactions (Figure S22). Although the BFE value indicates low affinity, this binding site includes the PHF6 region, which is essential for aggregation kinetics. Furthermore, the location of this binding site might be suitable for blocking the prion-like addition of Tau fibrils to the existing aggregate. Both the first and second poses in 5O3T were also observed in 6HRF (Figure S4), producing the two most negative docking scores (Table S2). While only docking scores are insufficient to validate these results, we have seen that the same binding site yields similar results between systems, and the GQDs, especially GQD28, do not tend to translate much during simulation. Thus, we predict that both favorable poses in 5O3T would be conserved in 6HRF.

**Figure 4:**
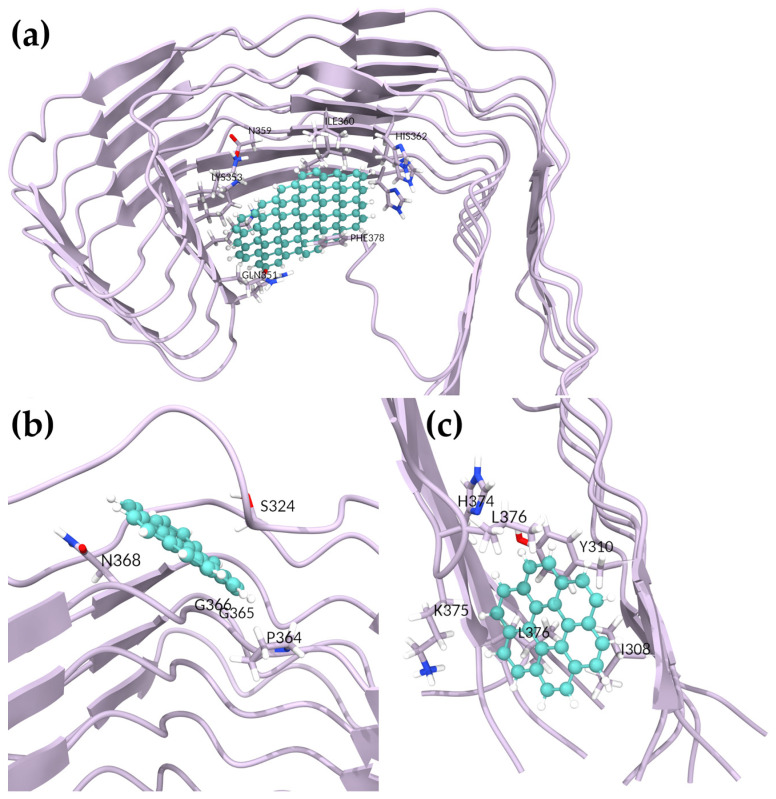
GQD binding sites in SF (5O3T) following MD simulations for (**a**) GQD28 Pose 1, (**b**) GQD7 Pose 1, and (**c**) GQD7 Pose 3.

To determine whether GQD7 would interact with 5O3T similarly, we simulated four GQD7-bound systems. The most negative BFE obtained was that of the third pose, with a BFE of −6.62 ± 3.72 kcal/mol, nearly twice the BFE of the other poses (Table 4). This pose was hydrophobic in nature, interacting most with I308 and Y310 of the PHF6 hexapeptide region (Figure 4 and Figure S23). This pose is particularly interesting since GQD7 was buried between the termini during the simulation, which was highly disruptive. Pose 1 showed interactions with S324, P364, G365, G366, and N368 and achieved a BFE of −5.81 ± 3.45 kcal/mol. Interestingly, the interactions formed by this pose were also observed in the GQD28 complex (Figure 4). The other two unique poses had similar BFEs of −3.97 ± 1.71 and −3.63 ± 2.54 kcal/mol (Poses 2 and 4, respectively). In summary, GQD7 exhibited favorable binding in two regions of the SF: L325, G326, and I328 (Pose 2) and V337, V339, K353, I354, G355, and L357 (Poses 4 and 5, respectively) (Figure S23). Given that negative docking scores were obtained in similar regions of the second SF structure (PDBID: 6HRF), it is expected that GQD7 would interact in a similar nature with 6HRF as with 5O3T, forming strong hydrophobic interactions with residues located in the 363–367 region of the structure.

Our analyses of two GQD sizes binding to SF shows that the larger size of GQD28 can alter the regions it interacts most strongly with. GQD28 forms the strongest interaction with the fluorescent SF binding site while GQD7 has two weaker interactions at this site. GQD28 forms specific interactions with SFs in this region, while GQD7 is more promiscuous and interacts strongly with the PHF6 region, which may be effective for aggregation prevention.

#### 2.6.2. Investigating GQDs Binding to Paired Helical Filaments

**Figure 5:**
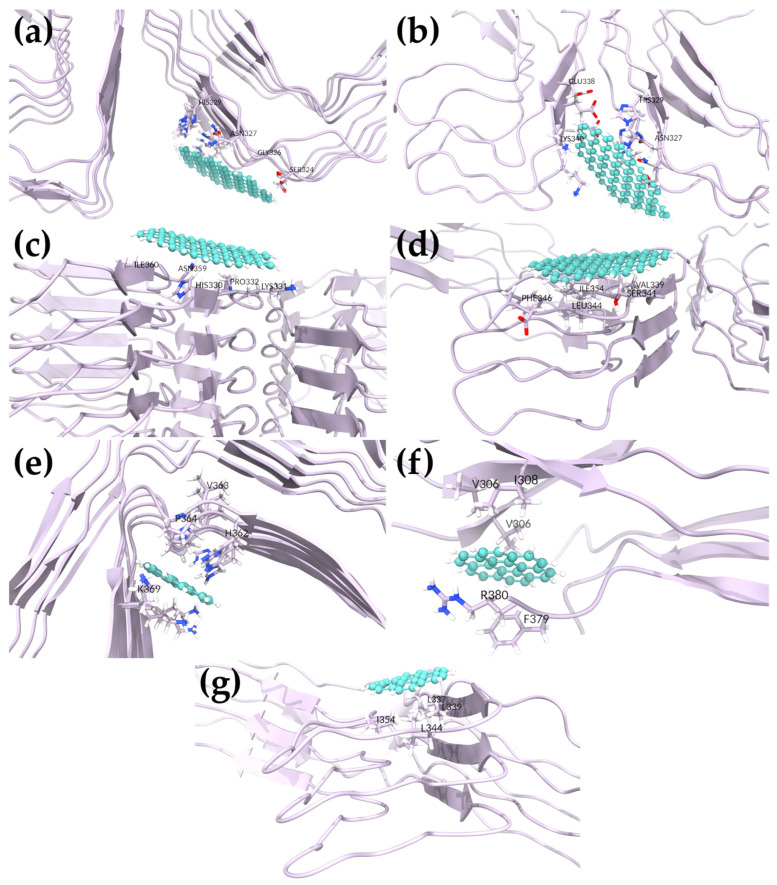
Favored binding sites in PHF (5O3L and 7YMN) structures: (**a**) GQD28 bound to 5O3L (Pose 2), (**b**) GQD28 bound to 7YMN (Pose 3), (**c**) GQD28 bound to 5O3L (Pose 6), (**d**) GQD28 bound to 7YMN (Pose 5), (**e**) GQD7 bound to 5O3L (Pose 2), and GQD7 bound to 7YMN in (**f**) Pose 1 and (**g**) Pose 3.

In the docking studies of 5O3L and 7YMN, GQD28 explored many binding sites that were not observed in the SFs. At the protofibril interface, both 5O3L and 7YMN had quite negative BFEs of −13.79 ± 4.38 and −8.31 ± 4.51 kcal/mol in Poses 2 and 3, respectively (Table 3, Figure 5). For 5O3L, GQD28 interacted most strongly with residues S324, G326, and N327 and had weaker interactions with I328 and H329 (Figure S24). For 7YMN, GQD28 interacted most strongly with residues N327, H329, and K340 and had weaker interactions with G342 and V339 (Figure S25). Interestingly, this region was recently shown to be the binding site of EGCG in vitro by trapping intermediates along the disaggregation pathway [39]. EGCG was capable of inducing sufficient conformational stress on PHFs to induce disaggregation. Given GQD28′s planar geometry, hydrophobicity, and similar interactions with Tau fibrils, it may similarly disrupt the fibrillar structure, as suggested in our stability analyses. Nevertheless, our present simulations cannot adequately investigate this.

Two similar binding sites were observed in both PHF structures above the protofibril interface. GQD28 had BFEs of −11.12 ± 3.26 and −12.02 ± 3.70 kcal/mol with 5O3L and 7YMN in Poses 6 and 5, respectively (Figure 5). For 5O3L, GQD28 interacted most strongly with H329, H330, L331, P332, and I360 (Figure S24). Despite being docked to a similar binding site, 7YMN translated during the simulation, interacting most strongly with V339, S341, L344, F346, and I354 (Figure S25). Though the interacting residues are slightly different, this region clearly has favorable binding and interactions with GQD28. We hypothesize that stronger binding to this region (e.g., through functionalization) could prevent the addition of Tau fibrils to a pre-existing aggregate and thereby inhibit the aggregate seeding process, a key molecular mechanism driving NFT formation. Further, GQD28 may also disrupt the interface of the protofibrils in this binding site which could lead to disaggregation.

For GQD7, two docking poses were selected with 5O3L and pursued for BFE determination. The first of these showed GQD7 binding to various regions of 5O3L, departing from its initial docked region. After 300 ns of MD, stable GQD7 binding to a specific region was not observed and this indicated that GQD7 can bind many more regions of Tau compared to GQD28. The second pose, however, proved to be stable (Figure 5) and yielded a BFE of −4.13 ± 2.45 kcal/mol, forming interactions with H362, V363, P364, K369 (Figure S26). Investigation of 7YMN produced six poses with negative BFEs. From this, it is clear that GQD7 is forming significant interactions in the PHF6, 330–340, 350–365, and 360–370 regions of the sequence, similarly to GQD28 (Figure S27). Pose 1 showed the strongest BFE with a value of −8.42 ± 3.97 kcal/mol. GQD7 formed hydrophobic as well as cation–Pi interactions with PHF6 residues V306, I308, and R379, respectively (Figure 5 and Figure S27). This pose is particularly interesting since GQD7 buried itself between the termini, indicating that it may be able to prevent the structurally required termini interactions. Poses 2, 3, and 4 showed consistent GQD7 binding to residues V339, L344, and I354, with a BFE of −3.78 ± 2.82 kcal/mol obtained for Pose 3 (Figure 5 and Figure S27). These poses are also consistent with the one stable pose obtained for 5O3L, further supporting the idea that GQD7 binds to these specific hydrophobic residues of PHFs. Furthermore, GQD7 and GQD28 were both observed interacting in the same region, which suggests the binding specificity. The difference in BFE between the two GQDs can be explained by the fact that GQD28 contains a bigger Pi system that enables its stronger hydrophobic and polar-induced interactions. Indeed, through the exhaustive docking and BFE analysis of both GQD28 and GQD7, greater BFEs were obtained for GQD28 over GQD7. Poses 5 and 6 obtained weak binding affinities, suggesting that these binding regions are likely to be unfavorable for GQD7 interactions. Although disaggregation potential was not investigated in this study, GQDs binding to the region containing residues V339, L344, and I354 and residues I360, H362, V363 in PHF and SF, respectively, supports the idea that these nanoparticles have the potential to be an AD theranostic.

## 3. Materials and Methods

### 3.1. GQD Preparation and Density Functional Theory (DFT) Calculations

We created two GQDs (Figure 1) in ChemDraw 16 software by PerkinElmer (Waltham, MA, USA) and converted them to PDB format using OpenBabel [40]. Molecular geometry optimizations were performed at the M06-2X/6-31G(d) level of theory using Gaussian 16 software [41]. The hybrid M06-2X functional (with 54% of Hartree–Fock exchange) has been frequently applied as an appropriate functional for the calculation of graphene and graphene-like systems [25,42,43,44]. Therefore, it was deemed suitable for the DFT calculations carried out in this work. Calculations were conducted using a polarizable continuum model (PCM), with water as the solvent to replicate physiological conditions as closely as possible.

### 3.2. Structure Selection

Currently, no complete structure of Tau exists because it is an IDP, making its structure incredibly challenging to resolve experimentally. To gain insights into the binding of GQDs to Tau, appropriate structures from databanks had to be selected. Most available structures only cover small sections of the sequence, mainly residues 265–380. For our study, we looked for structures related to AD in the Protein Data Bank [45] with the best resolution and the greatest number of resolved residues. The selected structures include monomeric Tau (2MZ7, 267–312) [46], PHF from an AD patient’s brain (5O3L, 306–379) [47], and SF from an AD patient’s brain (5O3T, 306–378) [47], among others.

### 3.3. Protein Structure Preparation

Protein structures were prepared using the Schrodinger protein prep wizard [48,49]. This program automates and integrates the most frequently used tools and techniques in structure preparation to turn a raw PDB structure into all-atom, fully prepared protein models. Briefly, the PDB files of the protein alone were imported, and hydrogens were added. Proper formal charge and force field treatment allow the accurate prediction of side chain protonation states at physiological pH (7.4) and the hydrogen bond network to be optimized, employing a systematic, cluster-based approach called PROPKA [50].

### 3.4. Extra Precision Glide Docking

Extra precision (XP) glide docking was carried out using Maestro software (version 13.5), generating 5 poses per docked ligand. An exhaustive multi-grid approach was used to scan all potential binding sites in each protein structure. Neighboring grids were built to overlap with each other. The grid box size was consistent for each GQD, being three times the radius of gyration (R_g_) of the docked GQD (roughly 15 and 32 Å for GQD7 and QGD28, respectively).

### 3.5. Molecular Dynamics (MD) Simulations of Consensus Docking Poses

To determine how the binding of the GQDs would behave in a more realistic scenario, we employed MD simulations. From the obtained docked poses, we chose to pursue only the most unique poses and those with the most negative docking scores. All docked complexes were prepared using tLeap in AMBER [51] and were simulated using the AMBERff14SB force field [52]. The ligand parameter and topology files were generated using Antechamber, and then the protein and ligand systems were solvated using a cubic TIP3P water box model with a distance of 15 Å calculated from the outermost residues. The system was then neutralized with counter ions (Na^+^ and Cl^−^) and brought up to a concentration of 0.15 M NaCl based on the number of water molecules. The systems were minimized for at least 10,000 cycles of the steepest descent algorithm. Following minimization, the systems were heated to 310.15 K at a constant volume using Langevin dynamics under periodic boundary conditions, with an NPT ensemble and a non-bonded cut-off distance of 12 Å, using the steepest descent algorithm. The systems were then equilibrated over multiple steps with decreasing constraints on the protein backbone and the GQD for around 10 ns, after which constraints on all atoms were removed to allow for the production stage of the simulation. All systems were simulated for 300 ns and extended to 340 if needed.

### 3.6. Data Analysis and Binding Free Energy Calculations

The trajectories were processed using CPPTRAJ in AMBER [53]. The VMD (Visual Molecular Dynamics) visualization program was then used for trajectory analysis [54]. ChimeraX 1.6 software was used for generating structure figures [55]. Using AMBER Tools [51], we calculated binding free energies (BFEs) with both molecular mechanics with generalized Born and surface area solvation (MM/GBSA) and molecular mechanics Poisson–Boltzmann surface area (MM/PBSA). MM/PBSA considers entropy, electrostatics, and solvation free energy, which will likely yield more realistic results than MM/GBSA, especially in solvent-exposed systems. The results obtained from these binding-free energy calculations in combination with the RMSD and DSSP of the systems allow for an extensive analysis of the GQD binding modes.

## 4. Conclusions

In this study, we exhaustively docked two different sizes of GQDs, GQD7 and GQD28, against many monomeric, SF, and PHF structures of Tau protein that are relevant to AD to determine their ability and preference to bind these structures. Following the prediction of binding sites, we performed classical molecular dynamics simulations of the selected poses and estimated the free energy of the binding of the GQDs in their dynamic complex environment. The introduction of GQDs into monomeric systems showed notable differences in the overall fluctuations and secondary structures, which suggests their potential role in changing the conformational dynamics of the Tau monomers. The differential behavior of GQDs, which showed larger RMSD fluctuations in SF and generally smaller RMSD fluctuations in PHFs, suggests that GQDs might have different impacts on different types of aggregates. Interestingly, the secondary structures of both Tau aggregates tended to lose beta sheet character in the binding site, which indicates that the proximal instability induced by GQDs may propagate through the fibril and cause disaggregation. Both GQDs interacted with the PHF6 hexapeptide region in Tau monomers; however, GQD7 was found to form a synergistic interaction with both PHF6* and PHF6 hexapeptide regions, which significantly improved GQD7′s BFE. Strong binding to the PHF6 hexapeptide region indicated that both GQDs may be able to prevent the aggregation-prone tendencies of this region. In Tau aggregates, the effect of different-sized GQDs was evident, with GQD7 behaving promiscuously in SFs, and GQD28 showing a clear preference to a binding site centered on I360, which has been known to bind fluorescent molecules that are used for Tau aggregate imaging. With PHFs, GQD28 formed strong interactions on the face of the protofibril interface and in between the protofibrils. The latter pose allows GQD28 to interact with a site that has been shown to be important to the EGCG-induced disaggregation of Tau. Conversely, GQD7 favors interacting with the PHF6 hexapeptide region in both SF and PHF structures. Our analyses have identified several key binding sites for GQDs in Tau, which may be used for the detection and prevention of Tau aggregates and potential sites of disaggregation.

## Figures and Tables

**Table 1:** Docking scores, binding free energies, and interacting residues for GQD28 with monomeric Tau.

Structure PDBID	Docked Region	XP Glide Docking Score (kcal/mol)	MM/GBSA Binding Free Energy (kcal/mol)	MM/PBSA Binding Free Energy (kcal/mol)	Interacting Residues
6GK8	59–67	4.23			
4FL5	212–217	4.89			
4TQE	225–230	5.48			
**2MZ7-1**	298–312	−0.60	−77.61 ± 4.59	−26.37 ± 4.83	V287, I297, I308, V309, Y310
**2MZ7-2**	285–312	−0.63	−50.48 ± 2.67	−17.54 ± 3.10	P301, V306, I308, V309
**5N5A-1**	261–272	−0.36	−45.80 ± 4.72	−13.16 ± 4.18	V256, I260, N265
5N5A-2	273–283	0.05			
5N5A-3	259–268	4.41			
**5N5B-1**	294–304	−0.51	−49.69 ± 3.00	−16.62 ± 2.84	I297, V306, I308, Y310
5N5B-2	302–312	0.09			
**5V5B-1**	275–281	1.10	−30.08 ± 3.71	−9.91 ± 3.18	Q276, I278, N279, L282
5V5B-2	276–281	0.89			
5V5C	275–280	6.90			
**3OVL**	306–311	−0.53	−29.93 ± 2.55	−12.65 ± 3.01	I308, V309, Y310
**4NP8**	306–311	−1.38	−25.49 ± 4.31	−8.01 ± 2.98	I308, V309, Y310
**5K7N**	306–311	−1.07	−19.24 ± 1.13	−7.46 ± 2.45	I308, V309, Y310
6DC8	404–408	N/A			
6H06	420–426	3.94			

**Bold** entries were selected for subsequent MD simulations.

**Table 2:** Docking scores, binding free energies, and interacting residues for GQD7 with monomeric Tau.

Structure PDBID	Docked Region	XP Glide Docking Score (kcal/mol)	MM/GBSA Binding Free Energy (kcal/mol)	MM/PBSA Binding Free Energy (kcal/mol)	Interacting Residues
6GK8	58–68	1.93			
4FL5	214–218	1.52			
4TQE	220–224	1.91			
**2MZ7-1**	275–280	−1.53	−28.68 ± 2.78	−12.76 ± 3.25	K274, Q276, I308
**2MZ7-2**	306–311	−1.40	−27.59 ± 2.93	−11.13 ± 3.18	K274, Q276, H299, I308, V309
**5N5A-1**	265–270	−1.32	−30.64 ± 3.37	−15.38 ± 3.70	I260, G261, L266
**5N5A-2**	281–288	−1.53	−27.13 ± 3.14	−9.40 ± 3.91	L266, V287
**5N5B-1**	295–299	−2.39	−23.29 ± 3.92	−9.32 ± 4.01	G292, V300, P301
**5N5B-2**	307–314	−2.02	−29.05 ± 4.14	−12.10 ± 3.53	V306, V309
**5V5B**	276–280	2.04	−17.89 ± 5.84	−7.28 ± 4.18	K274, V275, I277, K281, K282
5V5C	275–280	2.08			
**3OVL**	306–311	−1.51	−13.14 ± 2.57	−6.25 ± 2.98	I308, K311
**4NP8**	306–310	−1.22	−13.20±3.69	−3.66±2.45	V309, Y310, K311
**5K7N-1**	306–311	−1.71	−10.53 ± 3.40	−3.09 ± 2.34	Q307, I308, V309, K311
**5K7N-2**	306–311	−1.51	−13.14 ± 3.38	−4.59 ± 3.35	I308, V309, Y310
6DC8	404–408	−0.94			
6H06	422–426	1.98			

**Bold** entries were selected for subsequent MD simulations.

**Table 3:** Docking scores, binding free energies, and interacting residues for GQD28 with Tau aggregates.

Structure PDBID	Docked Region	XP Glide Docking Score (kcal/mol)	MM/GBSA Binding Free Energy (kcal/mol)	MM/PBSA Binding Free Energy (kcal/mol)	Interacting Residues
**5O3T-1**	351–362	−4.46	−64.52 ± 5.24	−10.76 ± 4.94	Q351, I360, L353, D358, F378
**5O3T-2**	306–311, 374–378	−3.13	−40.13 ± 6.87	−4.70 ± 4.43	Y310, H374, L376, T377, F378
**5O3T-3**	317–321, 315–319	−1.98	−40.32 ± 2.82	5.73 ± 6.38	V313, L315, L378
5O3T-4	368–375	7.45			
**5O3L-1**	361–372	−2.33	−53.12 ± 4.13	0.06 ± 4.39	R349, I371, E372, T373
**5O3L-2**	326–331, 338–341	−1.84	−49.15 ± 3.72	−13.79 ± 4.38	S324, G326, N327, I328, H329
**5O3L-3**	366–373	−0.27	−44.49 ± 3.58	−6.89 ± 4.96	L325, I328, H329, V363
**5O3L-4**	353–362	−4.74	−50.43 ± 5.51	0.89 ± 5.12	Q351, D358, I360,
**5O3L-5**	339–354	−0.52	−40.93 ± 3.24	−3.05 ± 4.05	V339, L344, F346, V350, I354
**5O3L-6**	355–361	−1.83	−46.58 ± 3.02	−11.12 ± 3.26	H329, H330, L331, P332, I360
5O3L-7	353–358	5.06			
**7YMN-1**	306–312, 374–378	−3.72	−23.46 ± 23.01	−5.02 ± 6.00	Y310, K375, L376, F378
**7YMN-2**	353–367	−1.28	−1.93 ± 9.57	−0.17 ± 2.76	D358, I360
**7YMN-3**	326–331	−1.42	−49.69 ± 4.16	−8.31 ± 4.51	N327, H329, L340
**7YMN-4**	353–361	−0.82	−28.02 ± 20.84	−3.73 ± 5.67	V339, E342, L344, F346, I354
**7YMN-5**	337–356	−1.85	−50.82 ± 5.64	−12.02 ± 3.70	V339, S341, L344, F346, I354
7YMN-6	349–360	3.40			
7YMN-7	329–334, 335–340	3.66			

**Bold** entries were selected for subsequent MD simulations.

**Table 4:** Docking scores, binding free energies, and interacting residues for GQD7 with Tau aggregates.

Structure PDBID	Docked Region	XP Glide Docking Score (kcal/mol)	MM/GBSA Binding Free Energy (kcal/mol)	MM/PBSA Binding Free Energy (kcal/mol)	Interacting Residues
**5O3T-1**	315–320	−1.66	−26.38 ± 2.67	−5.81 ± 3.45	S324, P364, G366, G367, N368
**5O3T-2**	315–320	−2.38	−19.74 ± 2.28	−3.97 ± 1.71	L325, G326, I328
**5O3T-3**	320–325, 360–369	−2.41	−24.63 ± 3.70	−6.62 ± 3.72	I308, Y310, H374
**5O3T-4**	339–344, 354	−1.08	−21.02 ± 2.85	−3.43 ± 2.89	V337, V339, K353, L357
**5O3T-5**	339–345	−1.15	−19.02 ± 2.05	−3.63 ± 2.54	V339, K353, G355, I354
**5O3L-1**	330–340	−1.78			
**5O3L-2**	362–369	−1.70	−24.29 ± 3.14	−4.13 ± 2.45	H362, V363, P364, K369
**7YMN-1**	304–306, 378,379	−1.79	−24.45 ± 4.30	−8.42 ± 3.97	V306, I308, R379
**7YMN-2**	320–325, 350–355	−1.58	−19.09 ± 2.54	−1.93 ± 1.12	V339, I354, G355
**7YMN-3**	339–341, 350–355	−1.90	−21.31 ± 1.93	−3.78 ± 2.82	V337, V339, L344, I354
**7YMN-4**	350–355	−1.11	−18.33 ± 3.02	−2.24 ± 1.21	V339, L344, I354
**7YMN-5**	328–330, 358–360	−2.49	−16.88 ± 2.48	−0.87 ± 2.65	D358, N359
**7YMN-6**	313–317, 370–375	−1.57	−12.03 ± 2.89	−0.53 ± 1.42	D314, L315

**Bold** entries were selected for subsequent MD simulations.

## Data Availability

Data available upon request.

## Supplementary Materials

The following supporting information can be downloaded at: https://www.mdpi.com/article/10.3390/ijms24119476/s1.
