# Supplementary material for: Investigating the Theranostic Potential of Graphene Quantum Dots in Alzheimer’s Disease
Source: Int J Mol Sci. 2023 May 30;24(11):9476. doi: 10.3390/ijms24119476 (PMC10253610; doi:10.3390/ijms24119476)
Supplement: Supplementary file 1 [file ijms-24-09476-s001.zip › ijms-2390790-supplementary.pdf]

Evaluating the theranostic potential of graphene quantum dots in Alzheimer's disease

Max Walton-Raaby, Riley Woods, and Subha Kalyaanamoorthy [1]

University of Waterloo, Department of Chemistry, Waterloo, ON, N2L 3G1, Canada

Electronic Supplementary Information

(27 pages)

Contents:

Figure S1. Structures of straight filaments (SFs) and paired helical filaments (PHFs) with their chains labelled.

Table S1. Selected PDB structures for docking investigation.

Figure S2. GQD consensus poses with of a) GQD28 and b) GQD7 with 5O3T.

Figure S3. GQD consensus poses with of GQD28 with a) 5O3L, b)7YMN, GQD7 with c) 5O3L, and d) 7YMN.

Figure S4. Consensus poses for a) GQD28 and b) GQD7 docked to 6HRF.

Table S2. Docked poses with negative docking scores for GQD28 with 6HRF.

Table S3. Docked poses with negative docking scores for GQD7 with 6HRF.

Figure S5. RMSDs of GQD28 bound to 5O3T, relative to the unbound protein.

Figure S6. RMSDs of GQD7 bound to 5O3T, relative to the unbound protein.

Figure S7. RMSDs of GQD28 bound to 5O3L, relative to the unbound protein.

Figure S8. RMSDs of GQD7 bound to 5O3L, relative to the unbound protein.

---

[1] Corresponding author e-mail: subhak@uwaterloo.ca

Figure S9. Secondary structures of 5O3T a) protein-only, b) GQD28-Pose 1 with chain labels interacting directly with GQD28 coloured in purple.

Figure S10. Secondary structures of 5O3L a) protein-only, b) GQD28-Pose 2 with chain labels interacting directly with GQD28 coloured in purple.

Figure S11. Secondary structures of 2MZ7 a) protein-only, b) GQD28-Pose 2, c) GQD7-Pose 1.

Figure S12. Secondary structure occurrence in 2MZ7; a) protein-only, b) GQD28 pose 1.

Figure S13. RMSDs for monomeric complexes with GQD28.

Figure S14. RMSDs for monomeric complexes with GQD7.

Figure S15. Secondary structure occurrence in 5O3T; a) protein-only, b) GQD28 pose 1.

Figure S16. RMSDs of GQD28 bound to 7YMN, relative to the unbound protein.

Figure S17. RMSDs of GQD7 bound to 7YMN, relative to the unbound protein.

Figure S18. Secondary structure occurrence in 5O3L; a) protein-only, b) GQD28 pose 1.

Figure S19. GQD28's binding site in 2MZ7 following MD simulation (pose 1).

Figure S20. Decomposition of energy terms (kcal/mol) in MM/PBSA calculations for GQD28 with monomeric Tau; a) 2MZ7 (pose 1), b) 2MZ7 (pose 2), c) 3OVL, d) 4NP8, e) 5K7N, f) 5N5A, g) 5N5B, h) 5V5B.

Figure S21. Decomposition of energy terms (kcal/mol) in MM/PBSA calculations for GQD7 with monomeric Tau; a) 2MZ7 (pose 1), b) 2MZ7 (pose2), c) 5N5A (pose 1), d) 5N5A (pose 2), e) 5N5B (pose 1), f) 5N5B (pose 2), g) 5V5B, h) 3OVL, i) 4NP8, j) 5K7N (pose 1), k) 5K7N (pose 2).

Figure S22. Decomposition of energy terms (kcal/mol) in MM/PBSA calculations for GQD28 with SF (5O3T); a) pose 1, b) pose 2.

Figure S23. Decomposition of energy terms (kcal/mol) in MM/PBSA calculations for GQD7 with SF TA (5O3T); a) pose 3, b) pose 1, c) pose 4.

Figure S24. Decomposition of energy terms (kcal/mol) in MM/PBSA calculations for GQD28 with PHF TAs (5O3L); a) pose 2, b) pose 6.

Figure S25. Decomposition of energy terms (kcal/mol) in MM/PBSA calculations for GQD28 with PHF TAs (7YMN); a) pose 3, b) pose 5.

Figure S26. Decomposition of energy terms (kcal/mol) in MM/PBSA calculations for GQD7 with PHF TAs (5O3L) pose 1.

Figure S27. Decomposition of energy terms (kcal/mol) in MM/PBSA calculations for GQD7 with PHF TAs (7YMN); a) pose 1 and b) pose 3.

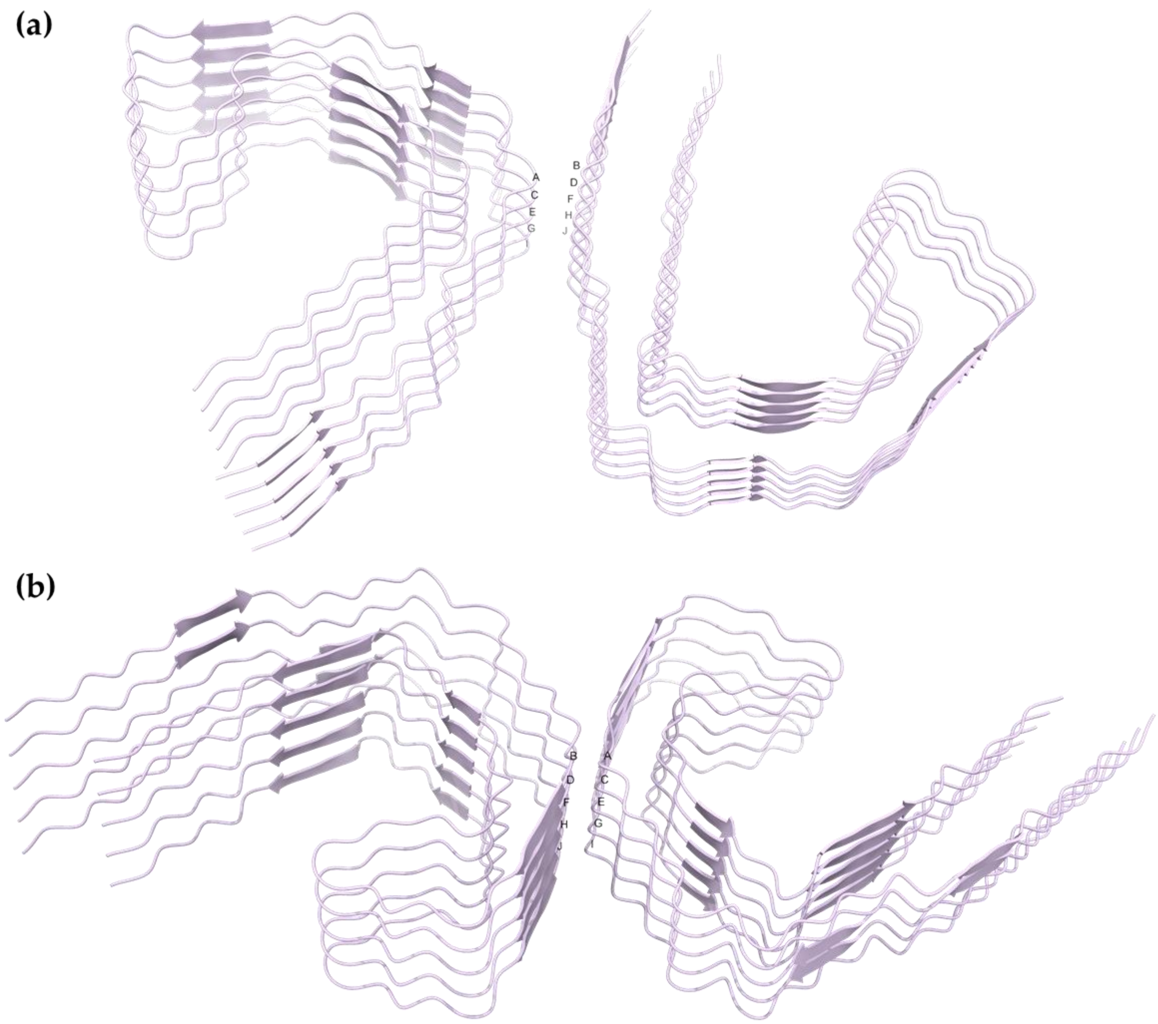

Figure S1. Structures of a) straight filaments (SFs) and b) paired helical filaments (PHFs) with their chains labelled.

Table S1. Selected PDB structures for docking investigation.

Region	NTD	PRR	R1	R2	R3	R4	CTD
Residues	1-151	151-244	244-275	275-306	306-337	337-369	369-441
PDB ID/seq coverage	6GK8/52-71	4FL5/210-219<br>4TQE/215-230	2MZ7/267-312<br>5N5A/254-290<br>5V5B/247-283	5V5C/275-280<br>5V5B/274-283<br>2MZ7/267-312<br>5N5A/254-290<br>5N5B/292-319<br>5O3T/306-378<br>5O3L/306-378<br>6HRF/304-380<br>7YMN/304-379	3OVL/306-311<br>4NP8/306-311<br>5O3T/306-378<br>5O3L/306-378<br>5K7N/306-311<br>2MZ7/267-312<br>5N5B/292-319<br>6HRF/304-380<br>7YMN/304-379	5O3T/306-378<br>5O3L/306-378<br>6HRF/304-380<br>7YMN/304-379	6DC8/404-408<br>6H06/420-426<br>5O3T/306-378<br>5O3L/306-378<br>6HRF/304-380<br>7YMN/304-379

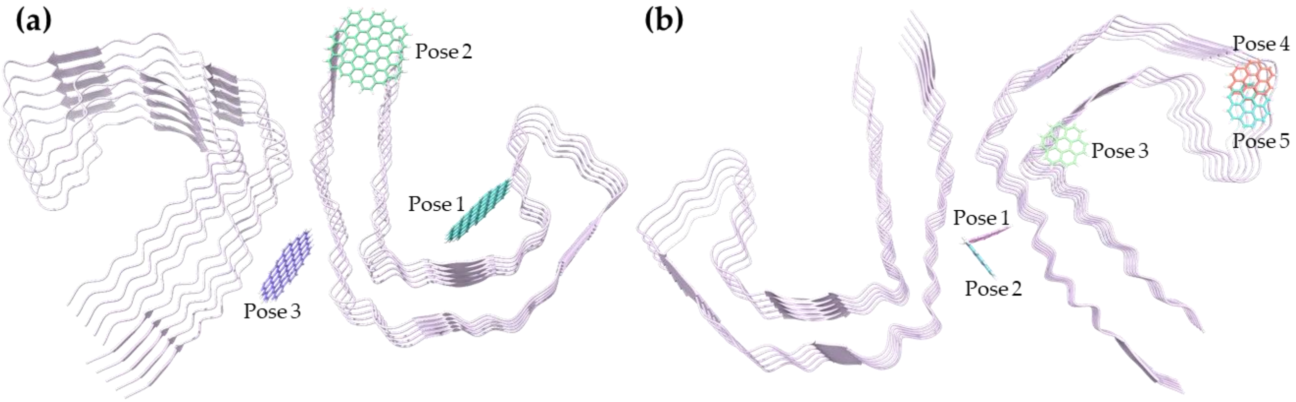

Figure S2. GQD consensus poses with of a) GQD28 and b) GQD7 with 5O3T.

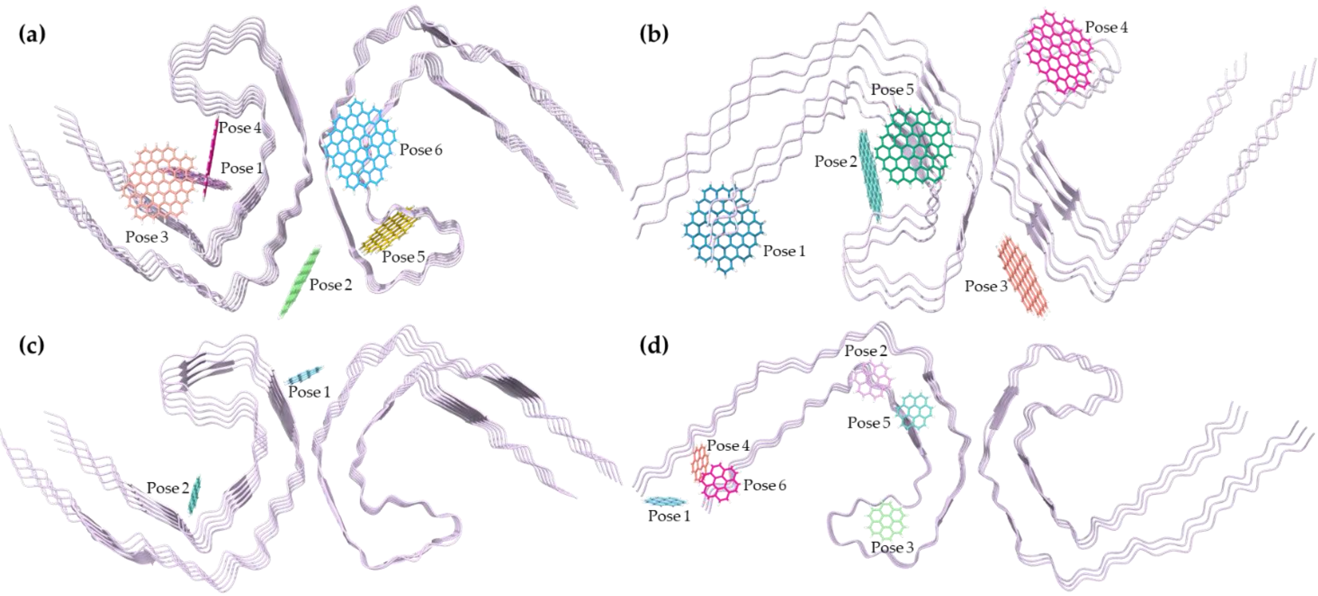

Figure S3. GQD consensus poses with of GQD28 with a) 5O3L and b) 7YMN, and GQD7 with c) 5O3L and d) 7YMN.

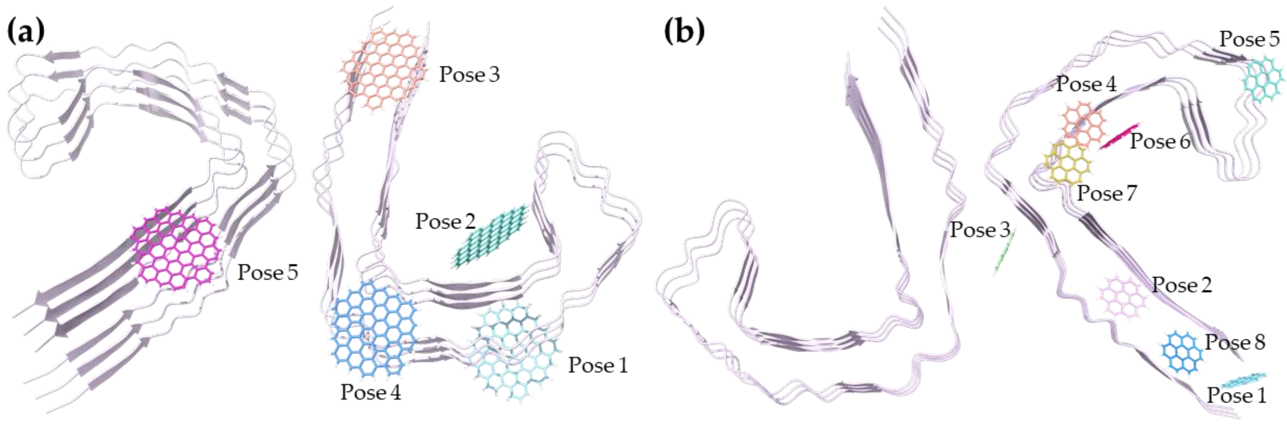

Figure S4. Consensus poses for a) GQD28 and b) GQD7 docked to 6HRF.

Table S2. Docked poses with negative docking scores for GQD28 with 6HRF.

Structure PDBID	Docked region	XP Glide docking score (kcal/mol)
6HRF-1	330-336, 355-360	-1.52
6HRF-2	351-362	-3.21
6HRF-3	306-311, 374-379	-2.07
6HRF-4	324-329, 361-365	-0.87
6HRF-5	311-317, 371-374	-1.25

Table S3. Docked poses with negative docking scores for GQD7 with 6HRF.

Structure PDBID	Docked region	XP Glide docking score (kcal/mol)
6HRF-1	304-306, 380	-2.23
6HRF-2	310-314, 370-375	-1.92
6HRF-3	317-321	-2.25
6HRF-4	360-365	-2.08
6HRF-5	340-346	-0.67
6HRF-6	358-363	-1.41
6HRF-7	320-325, 363-369	-1.84
6HRF-8	306-308, 380	-1.84

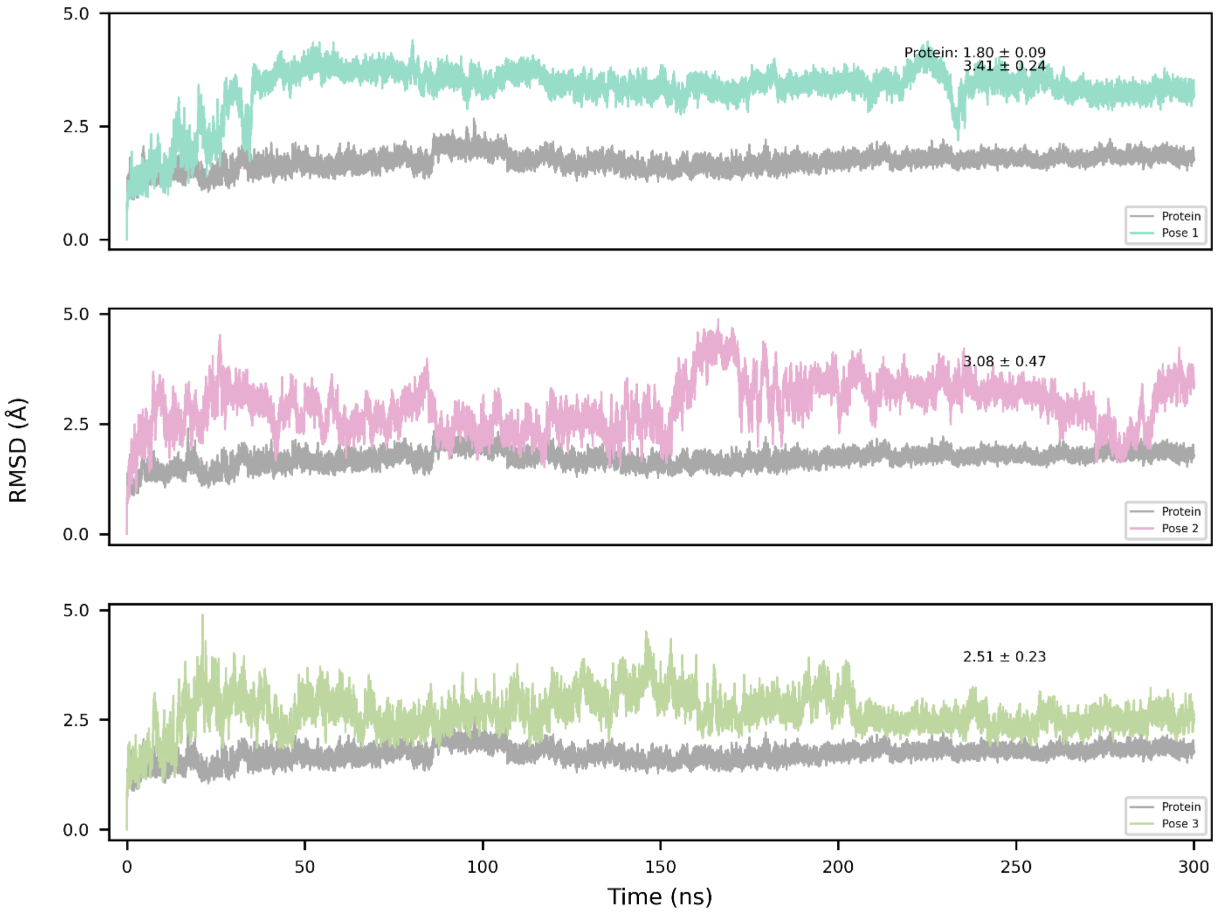

Figure S5. RMSDs of GQD28 bound to 5O3T, relative to the unbound protein.

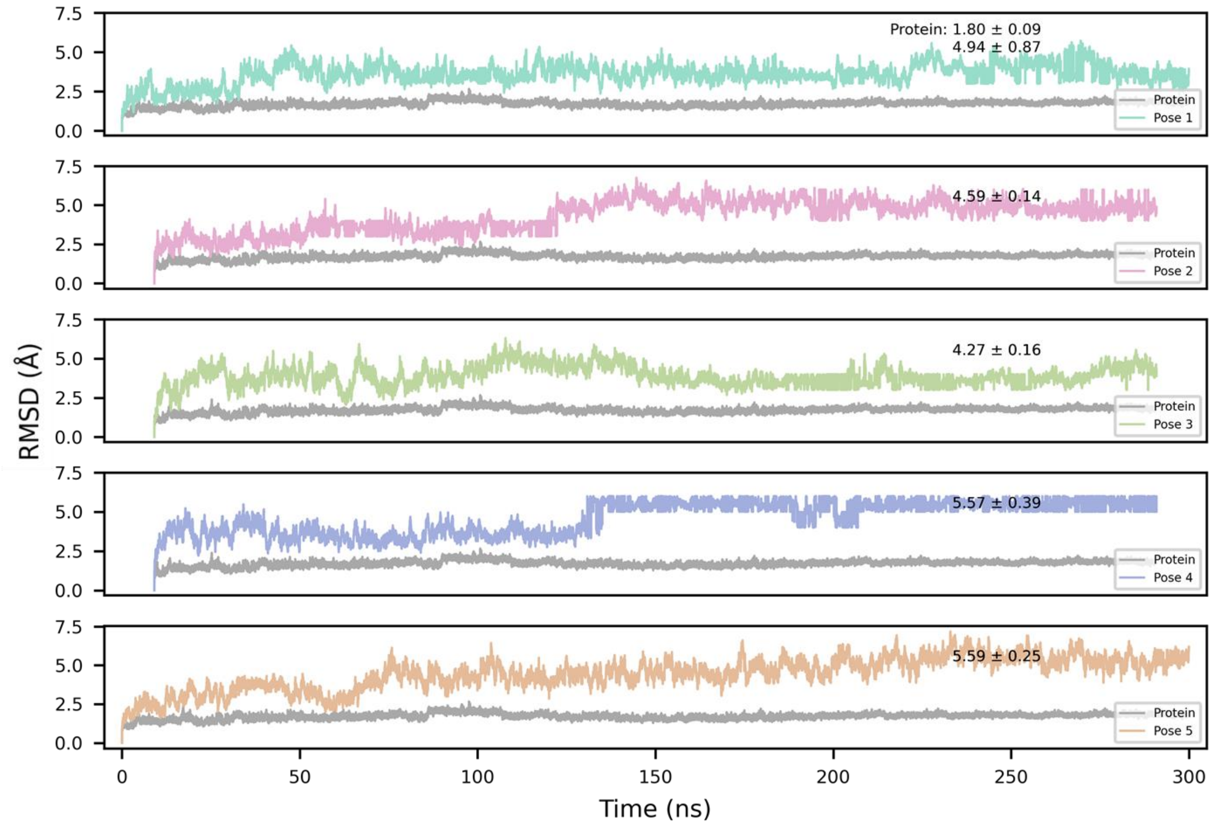

Figure S6. RMSDs of GQD7 bound to 5O3T, relative to the unbound protein.

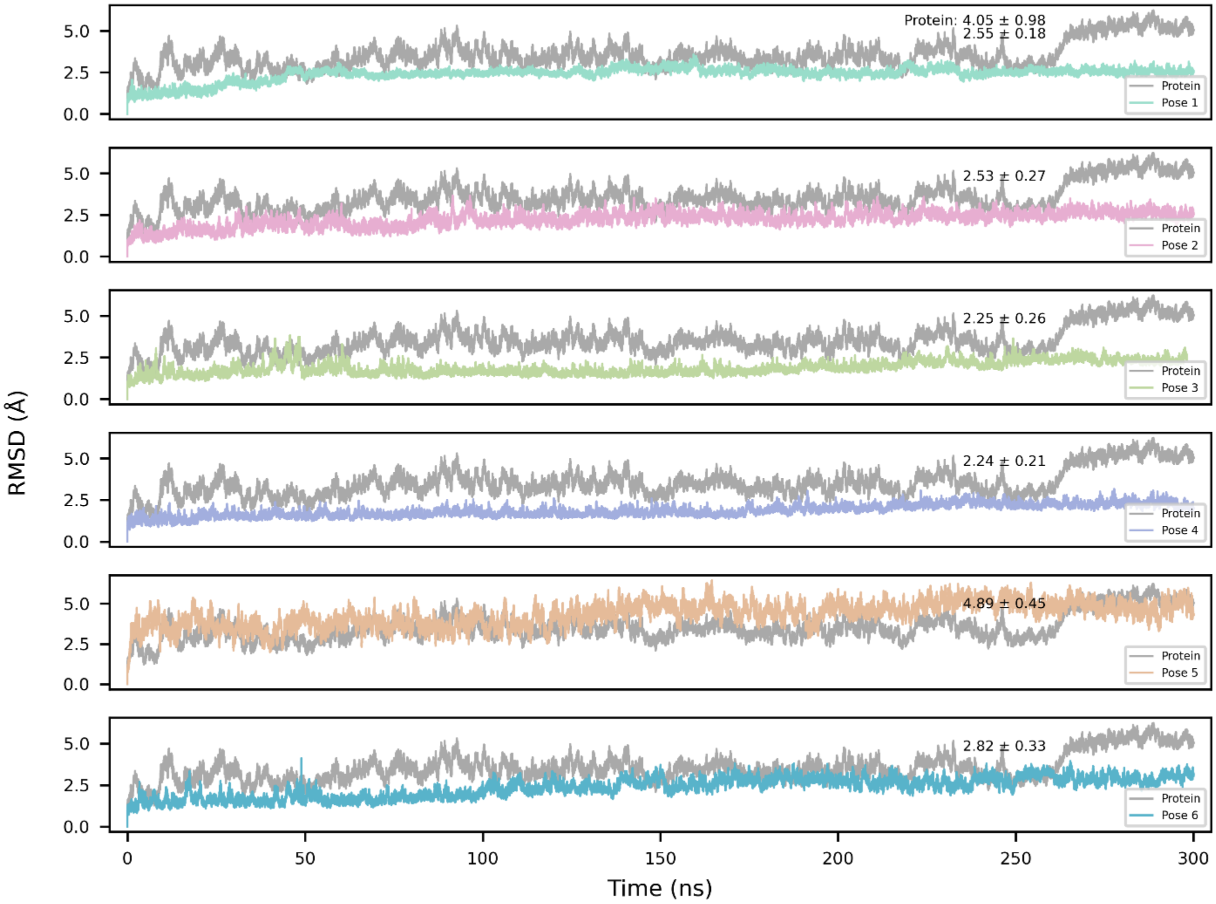

Figure S7. RMSDs of GQD28 bound to 5O3L, relative to the unbound protein.

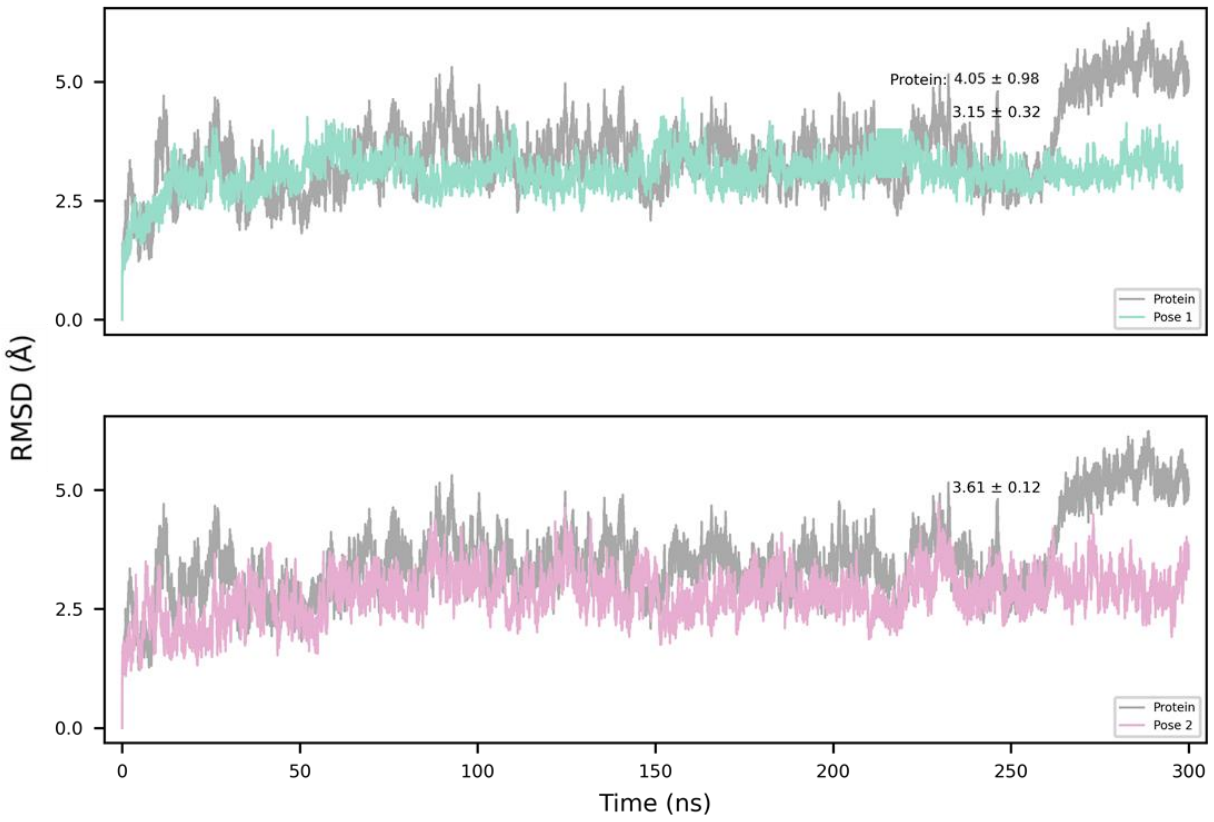

Figure S8. RMSDs of GQD7 bound to 5O3L, relative to the unbound protein.

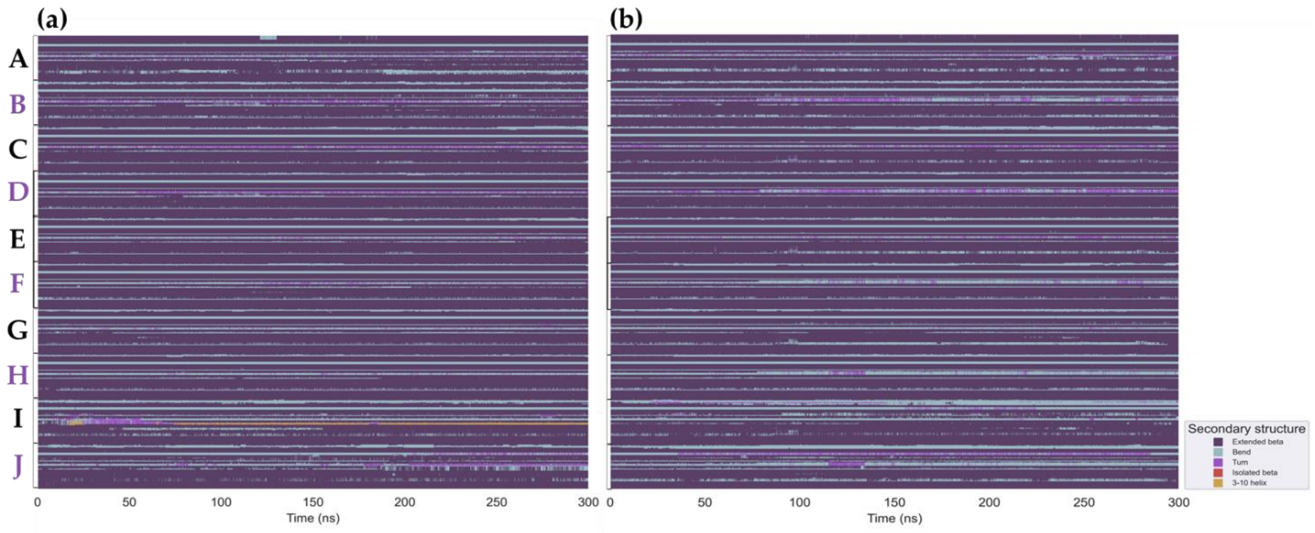

Figure S9. Secondary structures of 5O3T a) protein-only, b) GQD28-Pose 1 with chain labels interacting directly with GQD28 coloured in purple.

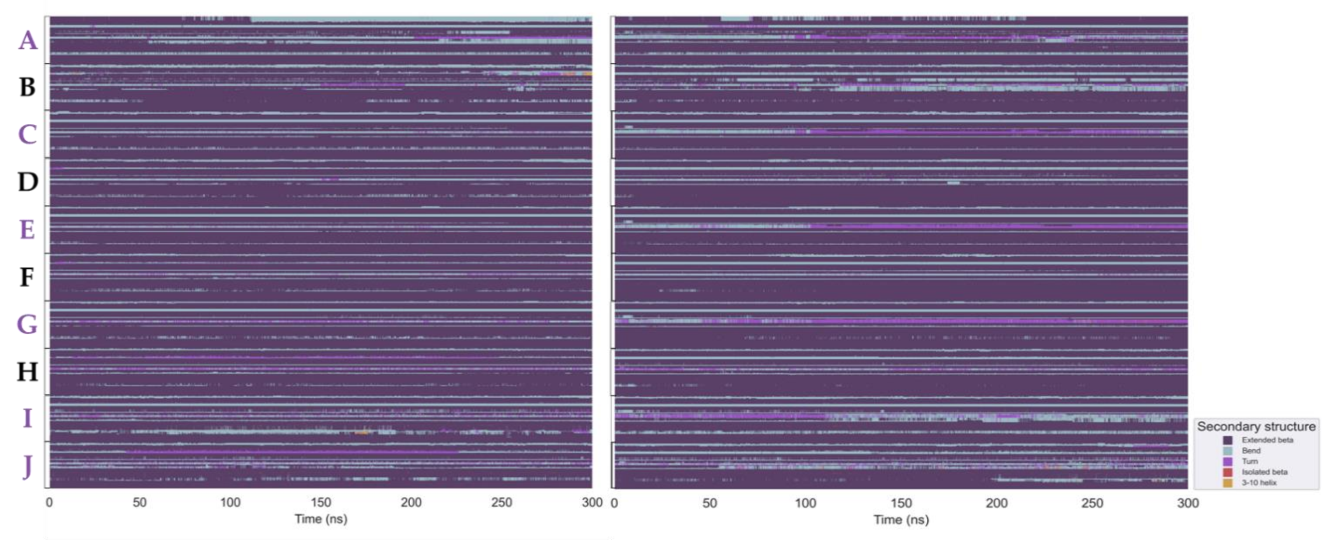

Figure S10. Secondary structures of 5O3L a) protein-only, b) GQD28-Pose 2 with chain labels interacting directly with GQD28 coloured in purple.

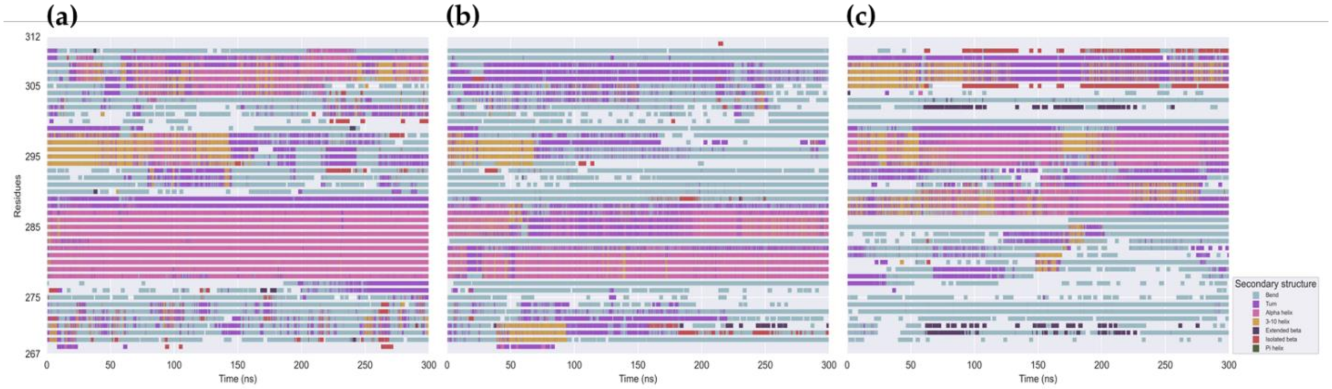

Figure S11. Secondary structures of 2MZ7 a) protein-only, b) GQD28-Pose 2, c) GQD7-Pose 1.

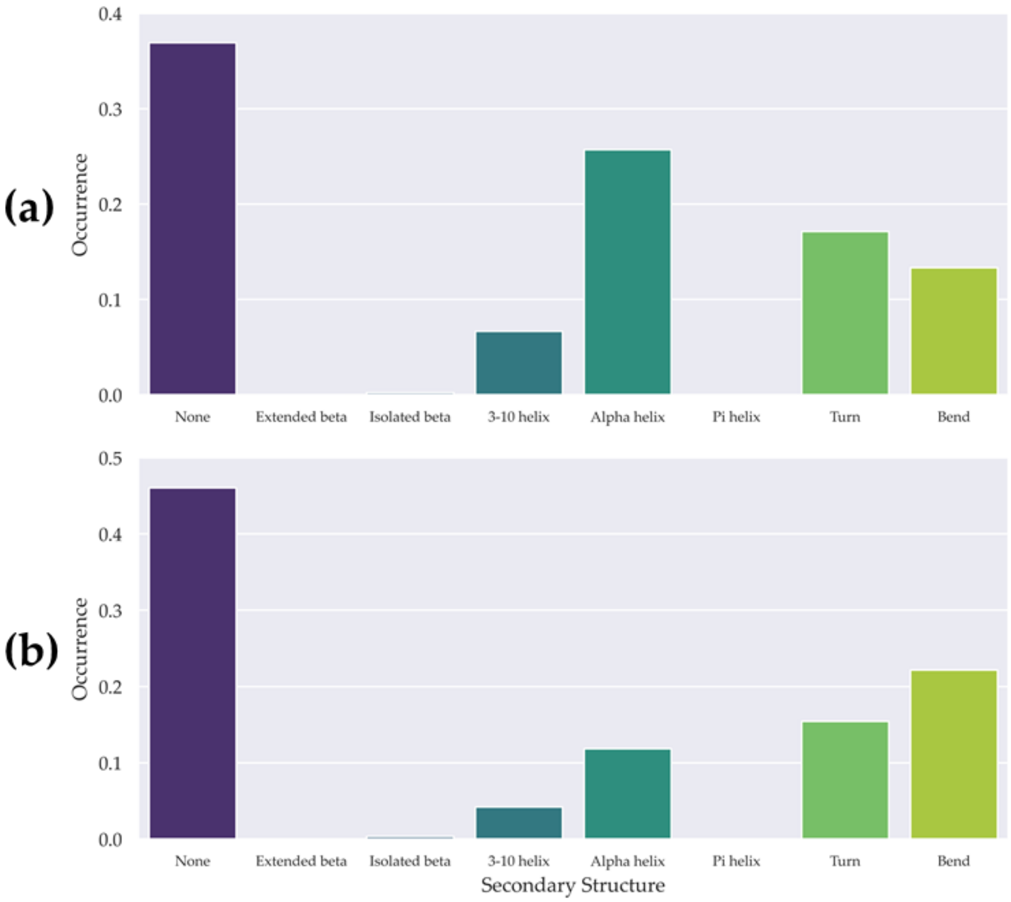

Figure S12. Secondary structure occurrence in 2MZ7; a) protein-only, b) GQD28 pose 1.

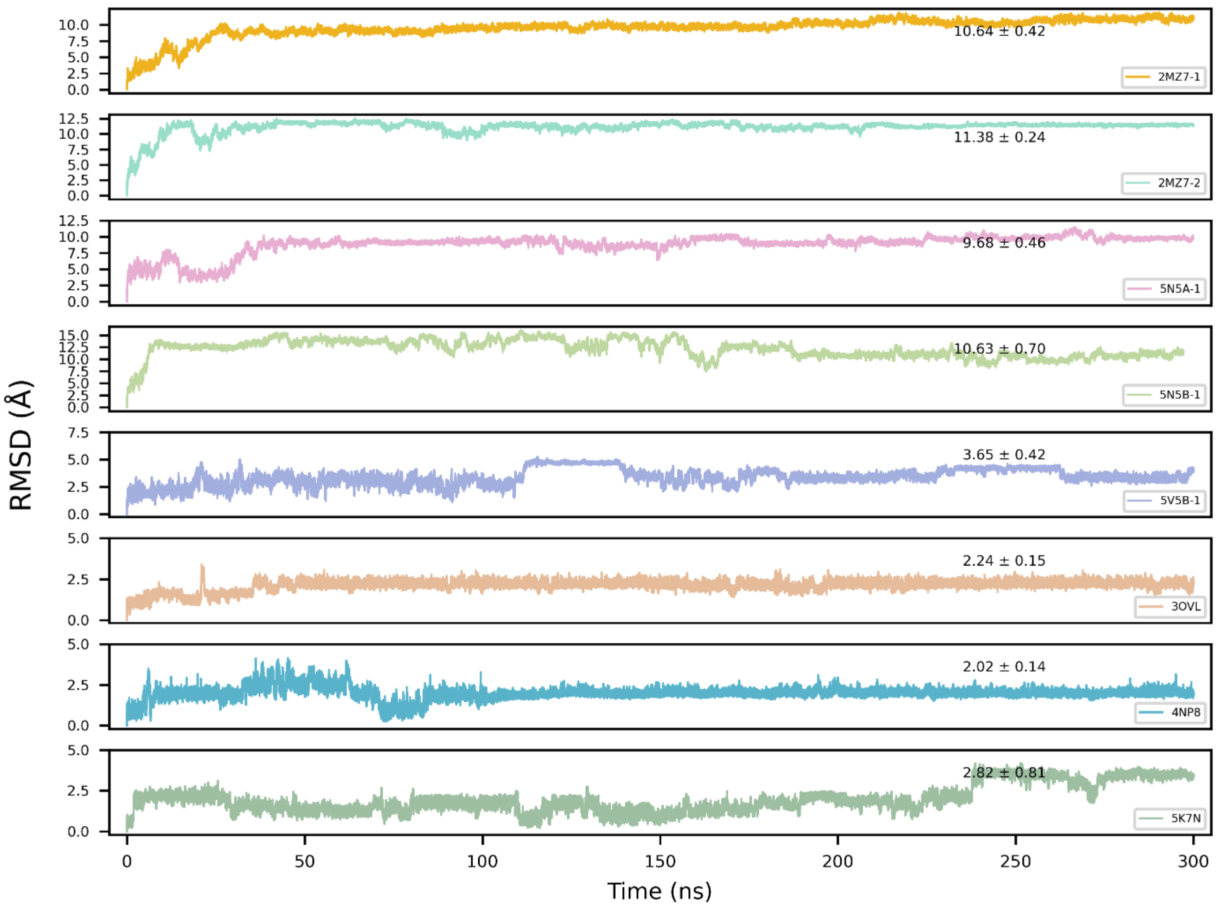

Figure S13. Root mean squared deviations for monomeric complexes with GQD28.

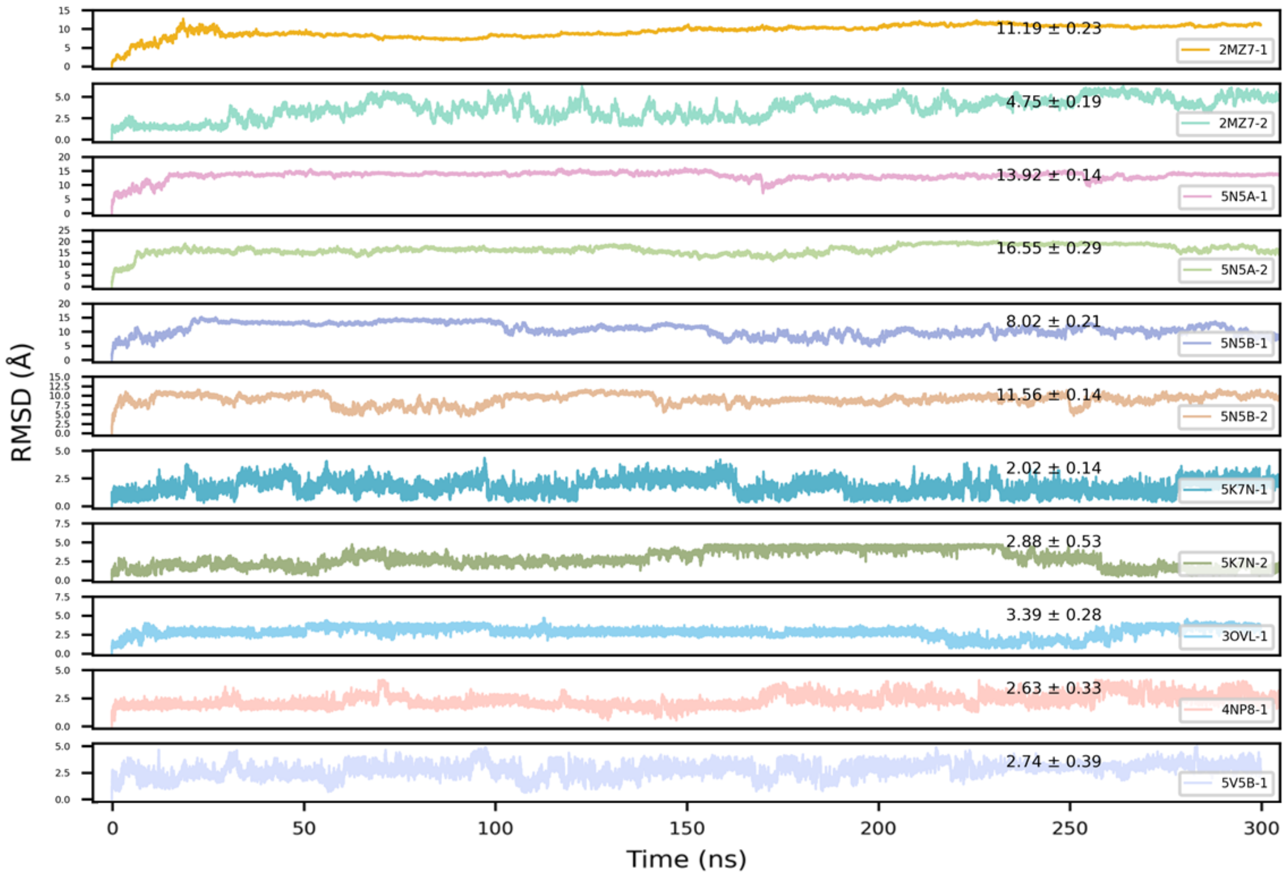

Figure S14. Root mean squared deviations for monomeric complexes with GQD7.

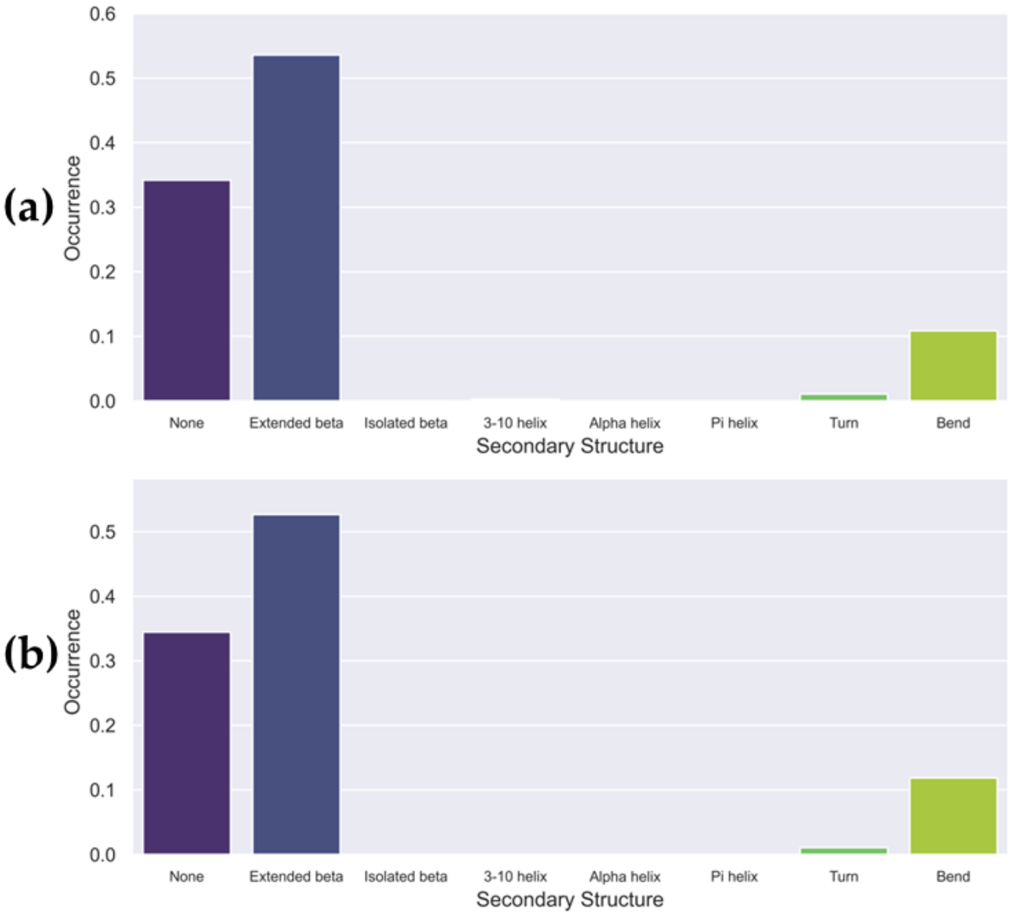

Figure S15. Secondary structure occurrence in 5O3T; a) protein-only, b) GQD28 pose 1.

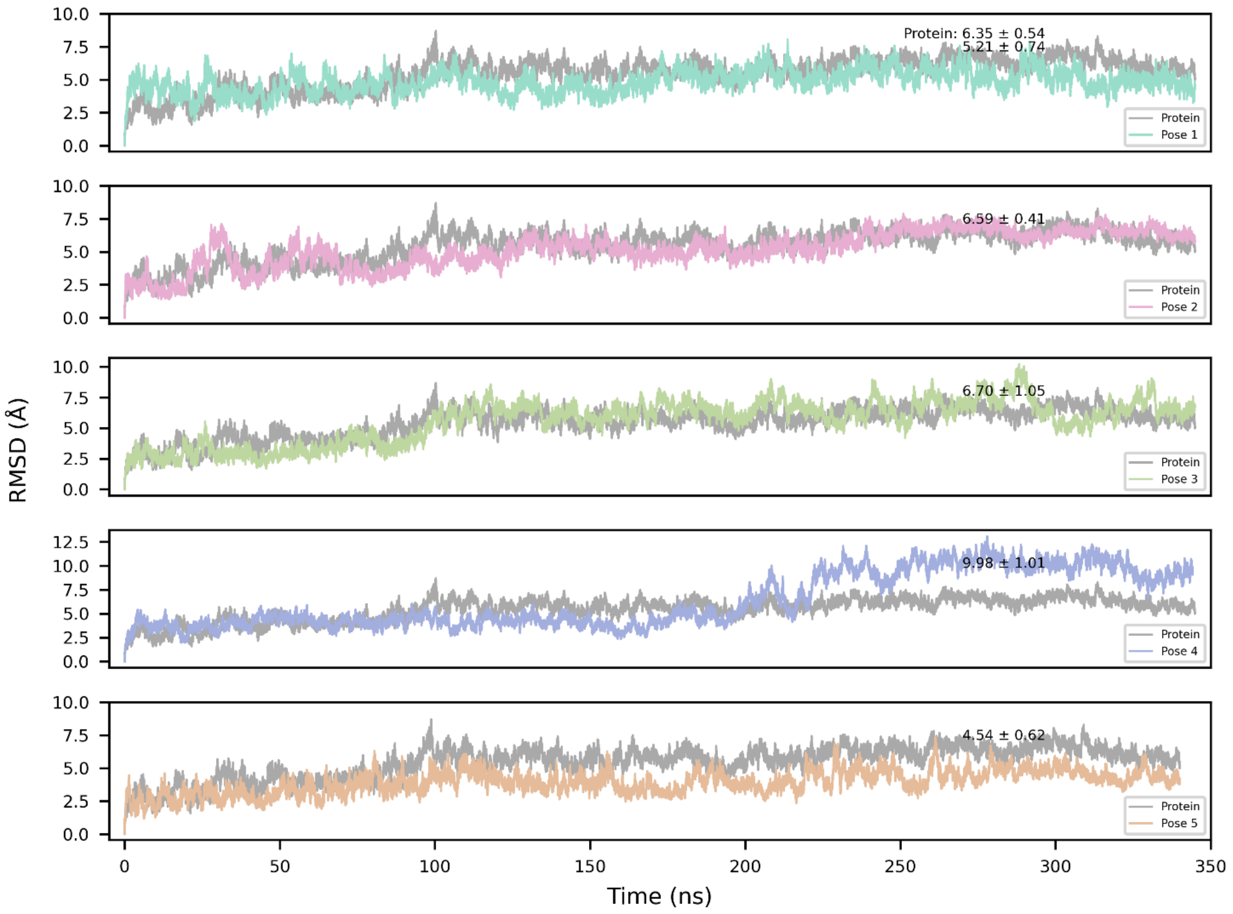

Figure S16. RMSDs of GQD28 bound to 7YMN, relative to the unbound protein.

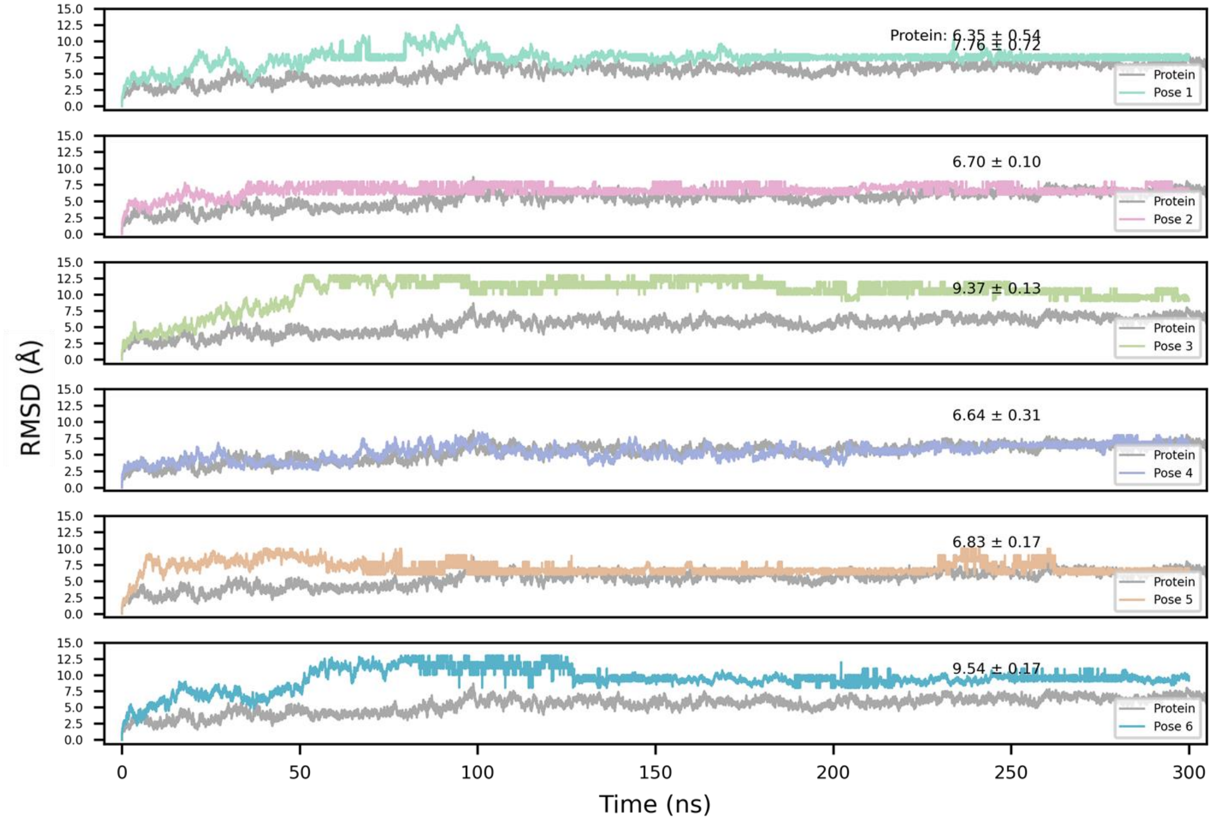

Figure S17. RMSDs of GQD7 bound to 7YMN, relative to the unbound protein.

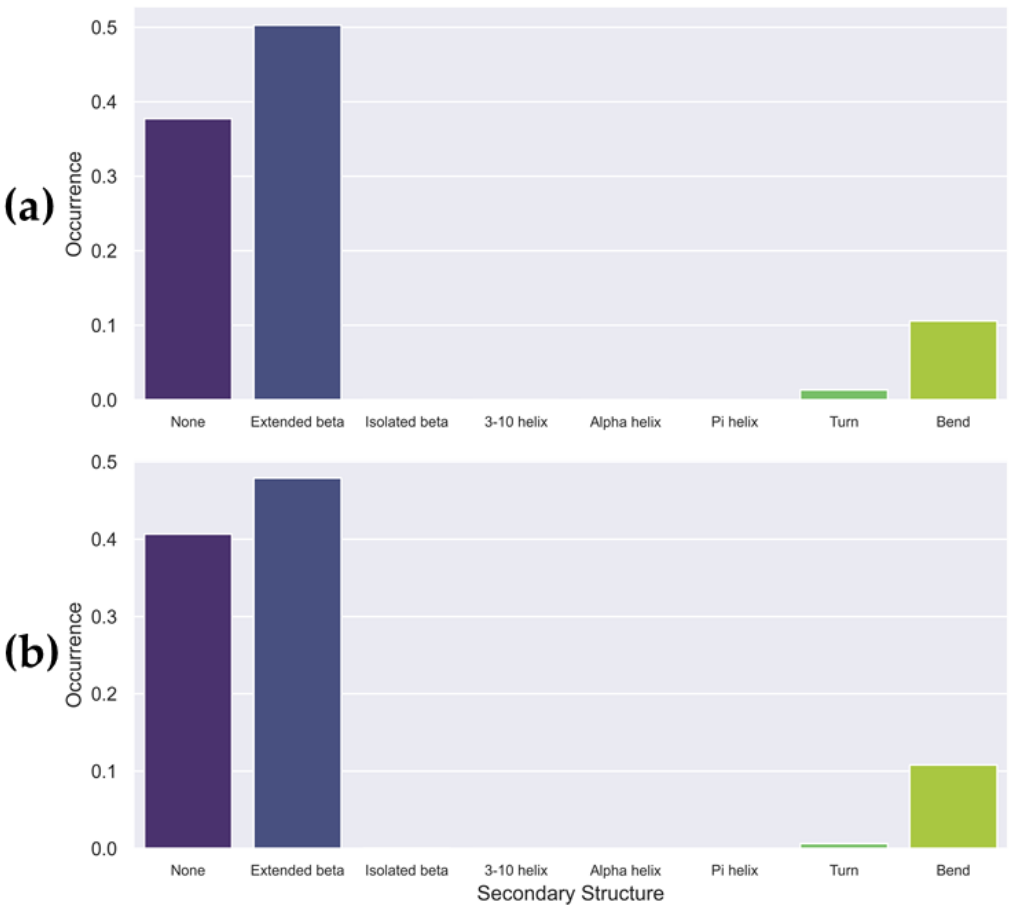

Figure S18. Secondary structure occurrence in 5O3L; a) protein-only, b) GQD28 pose 1.

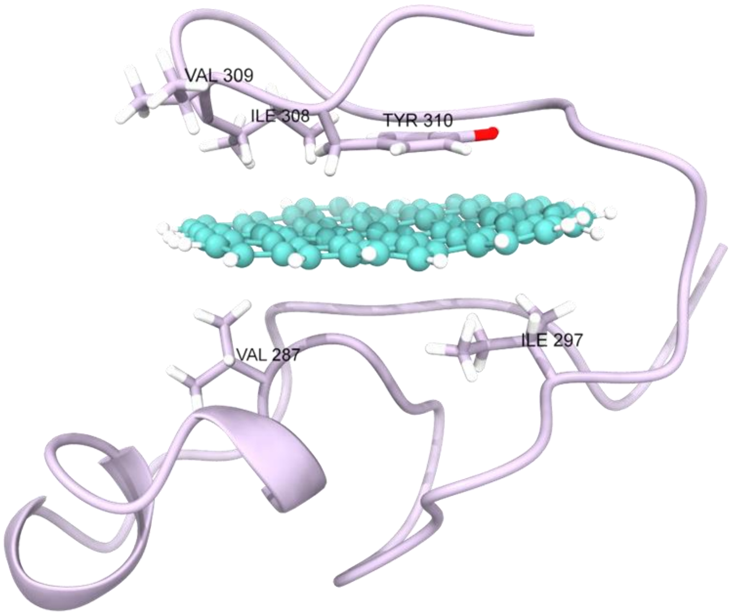

Figure S19. GQD28's binding site in 2MZ7 following MD simulation (pose 1).

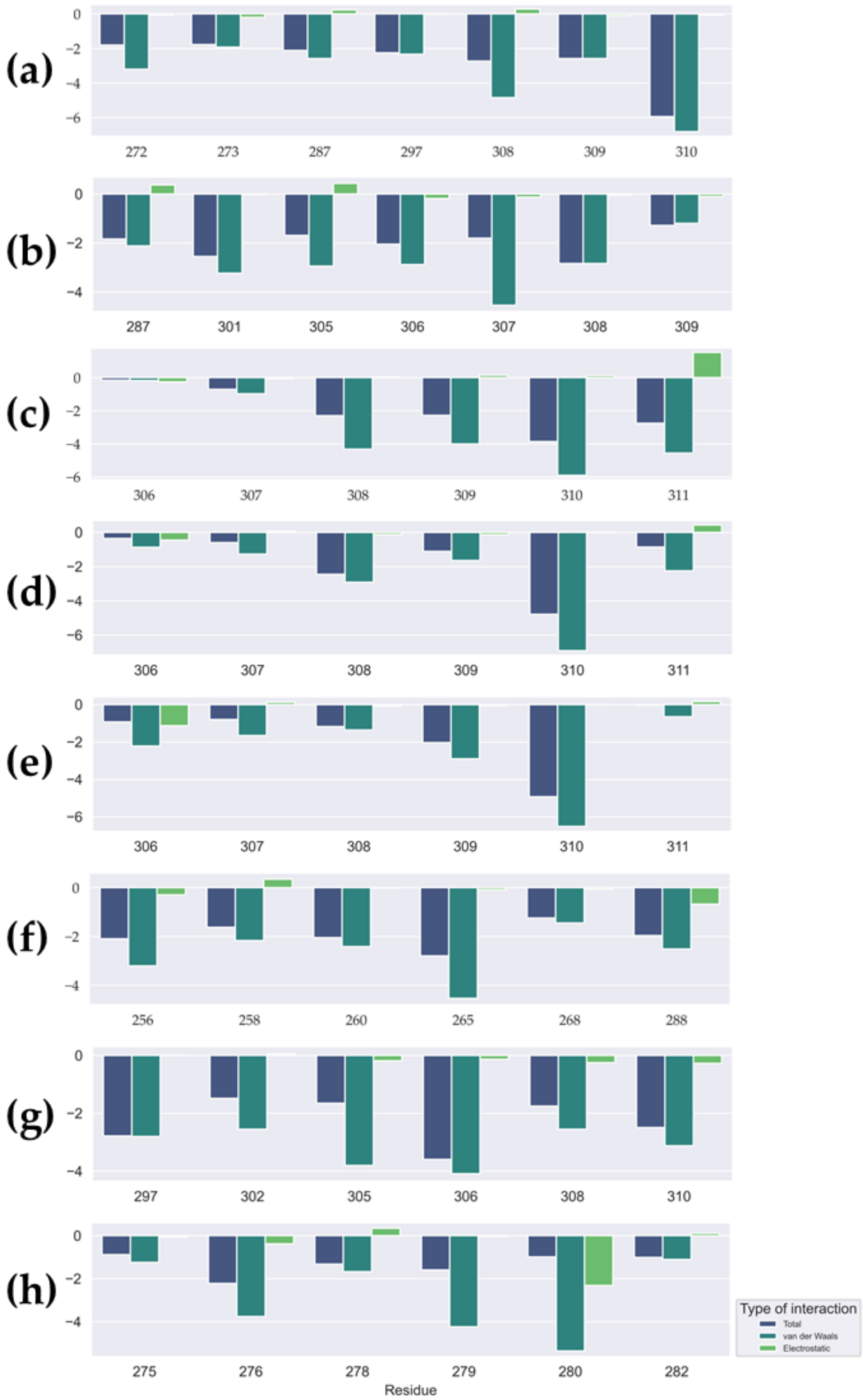

Figure S20. Decomposition of energy terms (kcal/mol) in MM/PBSA calculations for GQD28 with monomeric Tau; a) 2MZ7 (pose 1), b) 2MZ7 (pose 2), c) 3OVL, d) 4NP8, e) 5K7N, f) 5N5A, g) 5N5B, h) 5V5B.

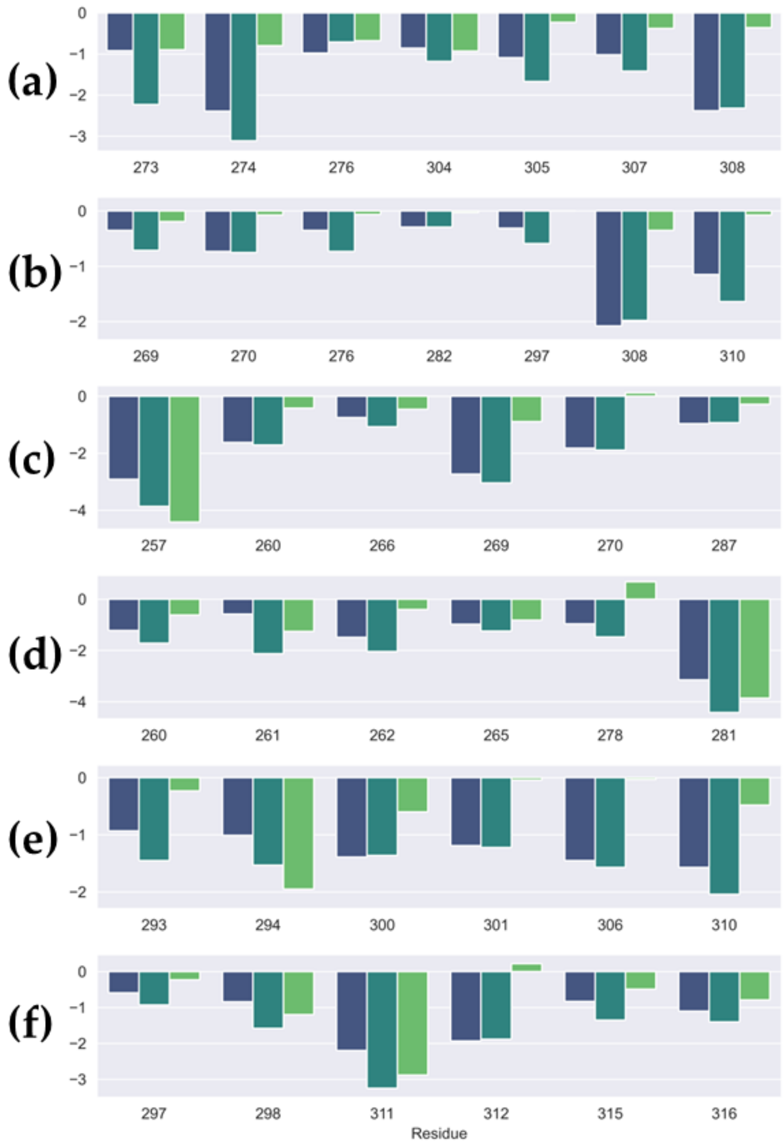

(a)
0
−1
−2
−3
273
274
276
304
305
307
308
(b)
0
−1
−2
269
270
276
282
297
308
310
(c)
0
−2
−4
257
260
266
269
270
287
(d)
0
−2
−4
260
261
262
265
278
281
(e)
0
−1
−2
293
294
300
301
306
310
(f)
0
−1
−2
−3
297
298
311
312
315
316
Residue

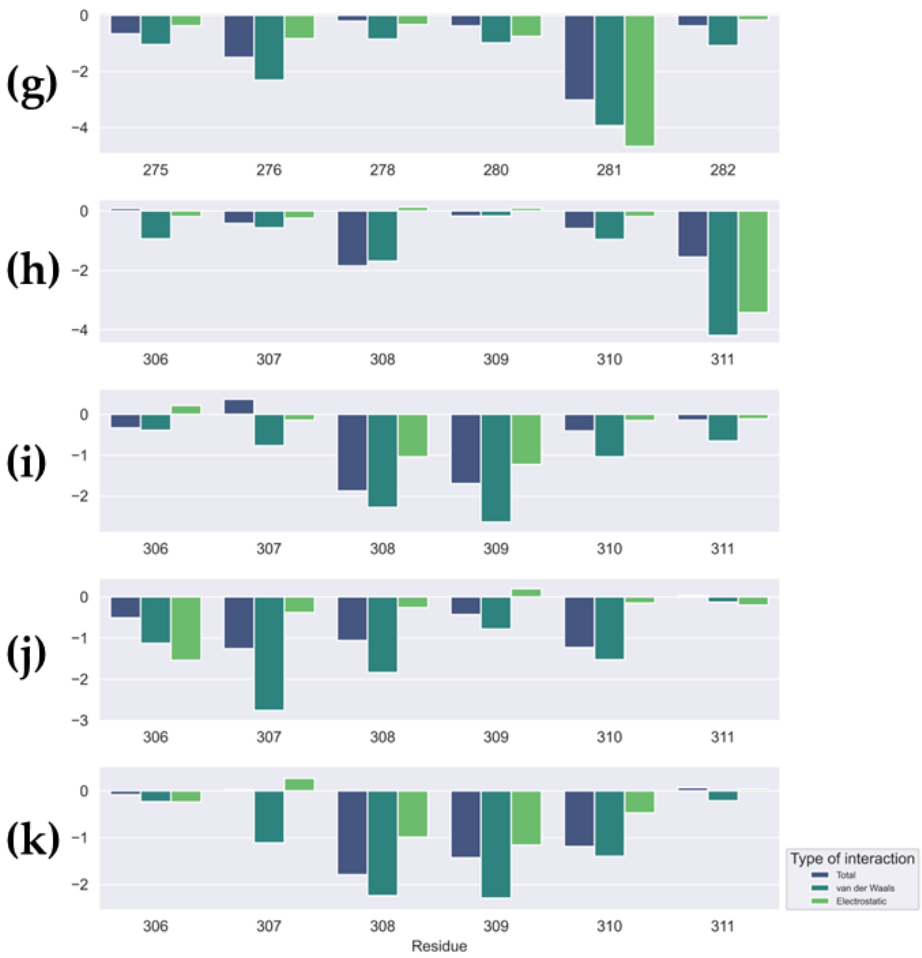

Figure S21. Decomposition of energy terms (kcal/mol) in MM/PBSA calculations for GQD7 with monomeric Tau; a) 2MZ7 (pose 1), b) 2MZ7 (pose2), c) 5N5A (pose 1), d) 5N5A (pose 2), e) 5N5B (pose 1), f) 5N5B (pose 2), g) 5V5B, h) 3OVL, i) 4NP8, j) 5K7N (pose 1), k) 5K7N (pose 2).

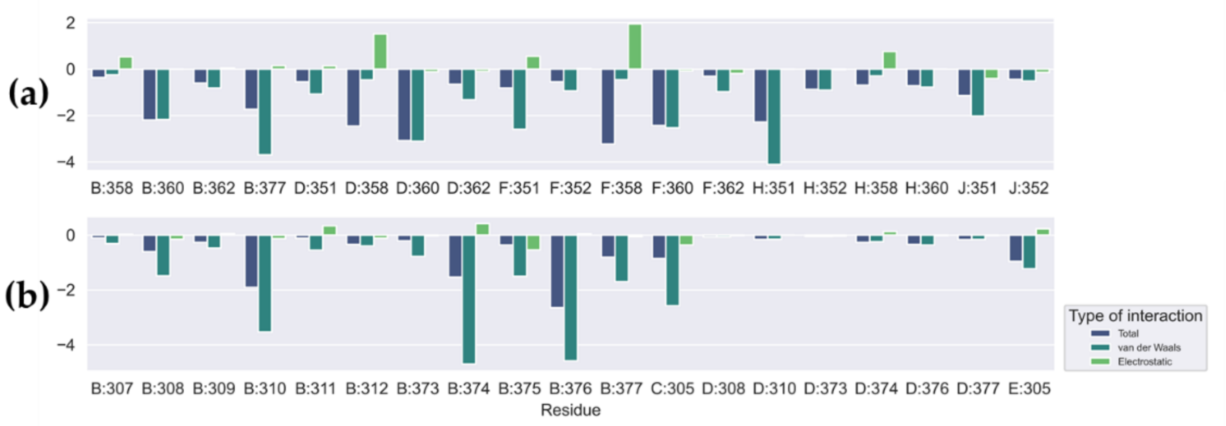

Figure S22. Decomposition of energy terms (kcal/mol) in MM/PBSA calculations for GQD28 with SF (5O3T); a) pose 1, b) pose 2.

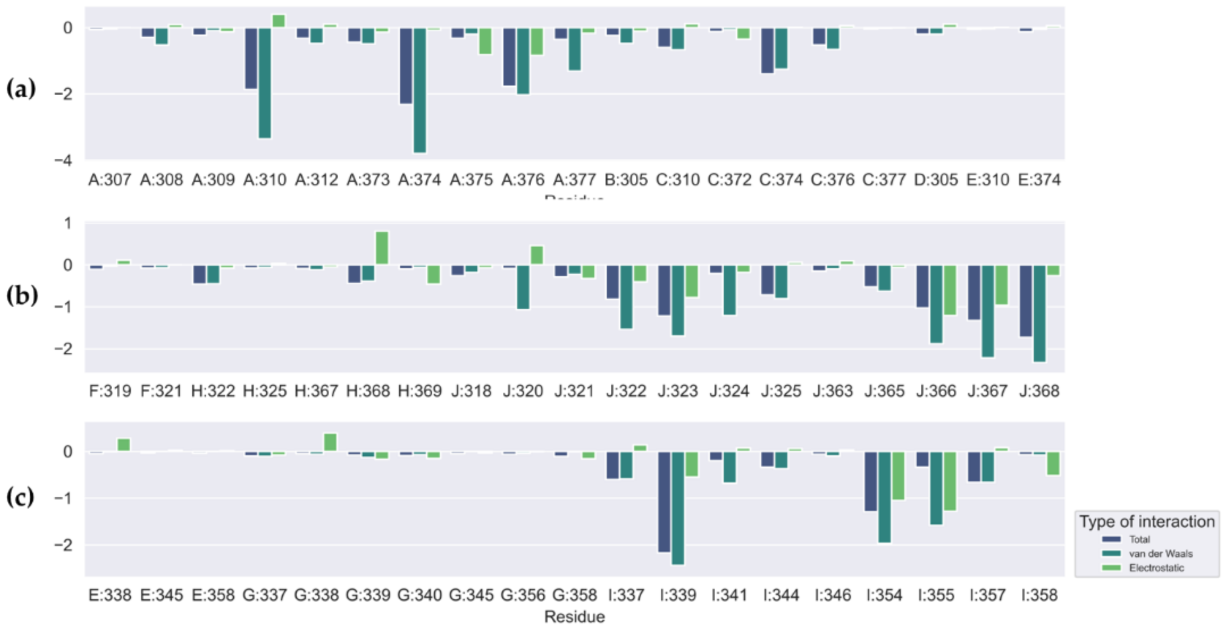

Figure S23. Decomposition of energy terms (kcal/mol) in MM/PBSA calculations for GQD7 with SF TA (5O3T); a) pose 3, b) pose 1, c) pose 4.

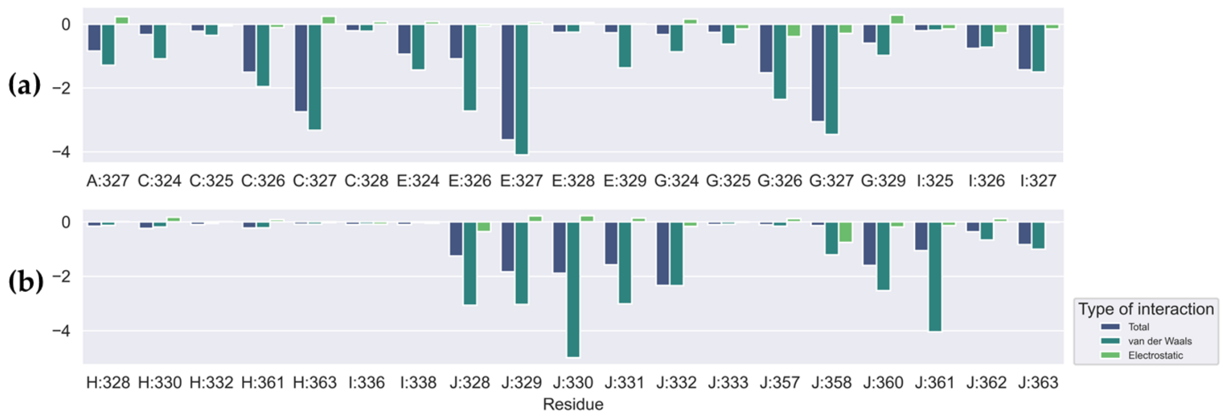

Figure S24. Decomposition of energy terms (kcal/mol) in MM/PBSA calculations for GQD28 with PHF TAs (5O3L); a) pose 2, b) pose 6.

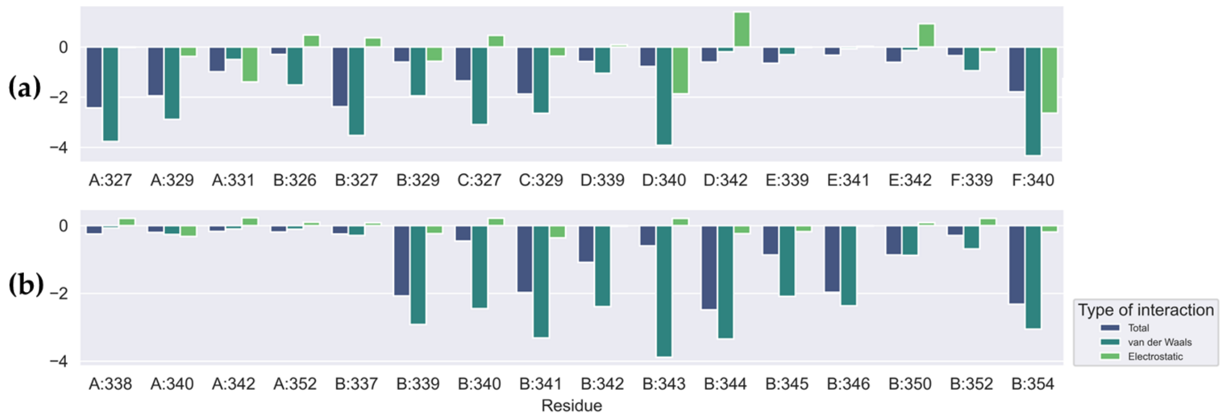

Figure S25. Decomposition of energy terms (kcal/mol) in MM/PBSA calculations for GQD28 with PHF TAs (7YMN); a) pose 3, b) pose 5.

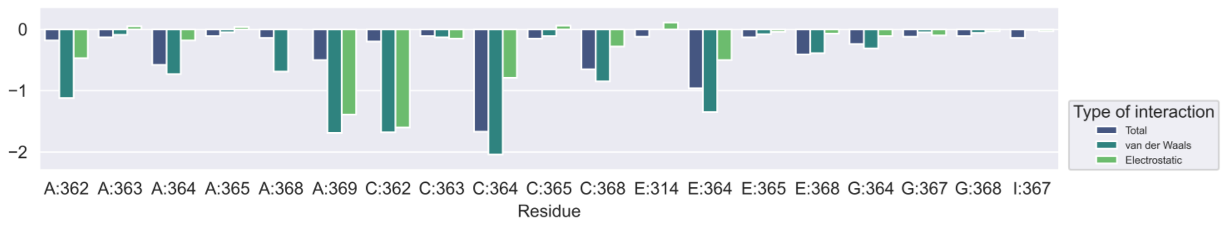

Figure S26. Decomposition of energy terms (kcal/mol) in MM/PBSA calculations for GQD7 with PHF TAs (5O3L) pose 2.

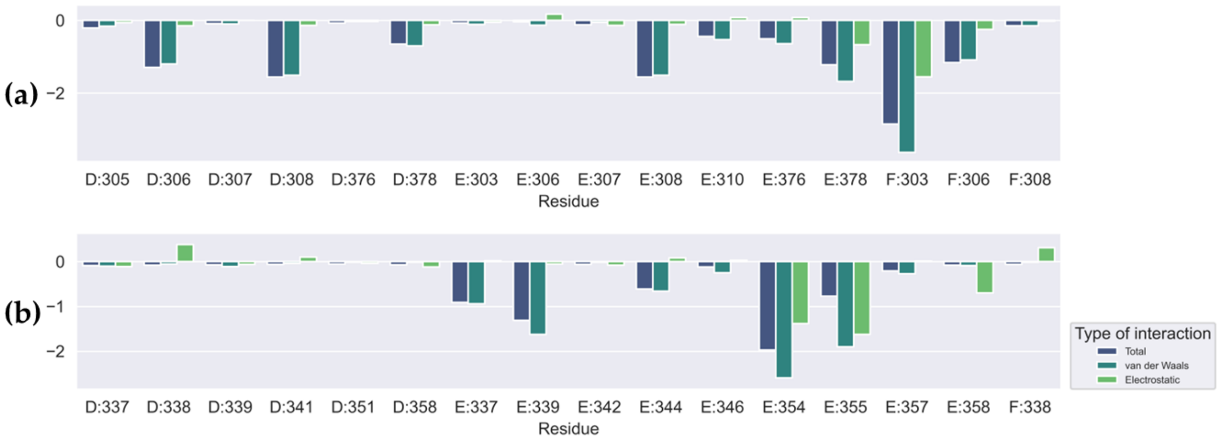

Figure S27. Decomposition of energy terms (kcal/mol) in MM/PBSA calculations for GQD7 with PHF TAs (7YMN); a) pose 1 and b) pose 3.
